# Microbially mediated silicon-based agro-wastes: a possible option in reducing bioaccumulation of arsenic

**DOI:** 10.3389/fnut.2025.1657640

**Published:** 2025-09-15

**Authors:** Sabyasachi Koley, Jancy Garg, Krisanu Golui, Amitava Rakshit

**Affiliations:** ^1^Department of Soil Science and Agricultural Chemistry, Institute of Agricultural Sciences, Banaras Hindu University, Varanasi, Uttar Pradesh, India; ^2^Department of Soil Science, Chaudhary Charan Singh Haryana Agricultural University, Hisar, Haryana, India

**Keywords:** arsenic, Si-based agro-wastes, microbes, transporters, dietary exposure

## Abstract

**Background:**

Arsenic (As), a class I carcinogen, affected 200 million people globally either through consumption of contaminated groundwater or food crops especially rice, leading to acute or chronic health issues including fatigue, respiratory diseases, liver fibrosis, and cancer.

**Research gap:**

For reclamation, majority of the efforts focused on single application of a particular amendment in reducing As levels in rice ecosystems.

**Methodology:**

This particular article comprehensively studied package of those amendments being used in reducing the bioaccumulation of As.

**Results:**

Consortia based package involving Si-rich agro-wastes (intact waste, compost, ash etc.) and agriculturally important microbes have the potential to reduce translocation of As to the above ground biomass by various mechanisms viz., competitive inhibition of transporters, iron plaque formation, anti-oxidant defense system, microbial oxidation etc. Rice straw compost (RSC) and husk composts (RHC) which are rich sources of Si (7–10%), Fe (700–900 ppm), Zn (40–60 ppm) and P (0.35–0.5%) have been explored owing the ability of Si and P to hinder the uptake of highly toxic As (III) and As (V) within plants by competitively inhibiting LSi1 and LSi2 for Si, and Pht4 and Pht8 transporters for P uptake with additional Fe released from amendments can form Fe-plaques that might work like As filters. Agro-wastes combined with silicate solubilizing bacteria significantly reduced As loading in final produce (25–52%), thereby reducing dietary exposure (ADI) even up to one third compared to control.

**Conclusion:**

This comprehensive review on understanding and validation of the mechanism provides a valuable insight in formulating a feasible As toxicity management strategy.

## 1 Introduction

Generation of agro-wastes is a ground reality by default with the extensive growth of agricultural productivity. The world population has been increased from 2.49 billion in 1950 to 8.19 billion in 2025. It is predicted to reach 9 billion by 2050 and to 11 billion by 2,100, respectively (1). Therefore, future food security poses a significant issue. There has been a dramatic increase in crop and livestock production to meet the intensive demands of a growing population, which has led to the formation of agro-wastes (2). Rapid population growth, economic prosperity, and an increase in agro-wastes production capacities have all been witnessed in Africa, China, and India within the past century (1). India produces around 850 Mt of agro-wastes annually which makes it the second largest producer of agro-wastes after China. Among the total agro-wastes generated by India larger portion are coming from paddy straw (130 Mt) (3). India's food grain production rose by 6% to a record 353.2 million ton (Mt) in the 2024–25 crop year (July–June) compared to previous year because of a significant rise in rice, wheat, pulses and oilseed output. This huge amount of production results in huge amount of waste materials. Additionally, there are public health concerns regarding the air pollution caused by the practice of burning rice residue, often known as parali (4). Greenhouse gases (GHGs) such as carbon dioxide (CO_2_), nitrous oxide (N_2_O), and methane (CH_4_) are produced when agricultural residue is not disposed of properly and are harmful to both humans and the environment (5). Whether it is waste material or a huge resource that is the main concern. The effective utilization of this vast volume of agricultural waste as a resource rather than a liability holds immense potential for advancing sustainable agriculture and contributing to societal wellbeing. Utilization or conversion of this huge resource is a tremendous challenge with a resultant impairment of natural resources due to unsustainable practices. The substantial generation of agro-wastes facilitates the reduction of heavy metal contamination in plants due to its intrinsic makeup. This review concentrates on arsenic among all heavy metals. Agro-wastes, particularly rice straw, rice husk, maize cob and sugarcane bagasse, possess a substantial amount of silicon. Utilizing this concentrated silica through the incorporation of agro-wastes into the soil might diminish arsenic bioaccumulation and enhance plant resilience against diverse biotic and abiotic stressors.

Several management strategies were proposed to maintain soil As bioavailability and grain As content below the recommended limits (6) as arsenic has impacted 200 million individuals worldwide from the ingestion of contaminated groundwater or food crops, particularly rice (7), resulting in acute or chronic health complications such as weakness, respiratory ailments, liver fibrosis, and cancer (8–11). However, majority of the efforts focused on single application of a particular amendment. Among different physical, chemical and biological remediation options implied, application of silicon (Si) emerged as a potential strategy in reducing the As load in grains (12, 13). Si and As(III) share the same transporters (Lsi1 and Lsi2) for their uptake and movement within the plant. Si can competitively inhibit the transporters and reduce the uptake of As. Application of Si can enhance the iron plaque formation and also reduce the conversion of short-range order ferrihydrite to goethite or siderite or other crystalline compounds of iron oxides or hydroxides present in Fe-plaque. Application of Si facilitate rhizosphere oxygenation by enhancing the radial oxygen loss which in turn induces microbial oxidation of Fe^2+^ to Fe^3+^ leads to more formation of Fe-plaque around the roots (14). This Fe-plaque has the potential of trapping As by adsorption or co-precipitation mechanism (15). A lot of studies described that application of inorganic silica sources (CaSiO_3_, NaSiO_3_, Si nano-particles etc.) reduced mobility of As from soil to plant. But exploring Si-based agro-wastes as a potential source of Si is rare and not exclusively studied. Moreover, these wastes are a rich source of iron, zinc, carbon, cellulose, lignin, and various inorganic or organic chemicals. These compounds play a specific role in restricting arsenic absorption.

Co-application of these Si-rich agro-wastes with silicate solubilizing microbes (SSM) can open a new path in reducing the As loading in final produce, thereby reducing dietary exposure up to one third compared to control. Si is abundantly available in earth crust (27.06% by weight) but often insufficiently available for crops, as plants generally uptake Si as monosilicic acid (H_4_SiO_4_). Higher plants especially rice removes Si rapidly, requires its supplementation. Although aqua soluble silica fertilizers like CaSiO_3_, NaSiO_3_, Si nano-particles provides large amount of Si, it can present a cost challenge for conventional agricultural practices. There is rare occurrence of negative effect of Si-fertilizer application (16). In light of high cost of inorganic Si-fertilizers, there is a much need of thinking viable, sustainable alternative strategies to address the issue of remediating As bioaccumulation. Application of Si-rich agro-wastes already resulted in a reduction of 20–40% of As concentration in rice grains (10). The potential of resistant SSM presents a practical, ecological, sustainable, and economical method to increase Si availability for crops by affecting the complex process of Si cycling. The solubilization of silica has been enhanced by SSM by many methods, including the formation of organic and inorganic acids, extracellular polysaccharides, ligands, or via nucleophilic assault. The microorganisms facilitate the solubilization of potassium (K) and Si, rendering them a viable alternative for bio-fertilization and potentially reducing reliance on synthetic fertilizers. The function of As-resistant silicate solubilizing bacteria (SSB) in reducing As uptake by rice necessitates further exploration, despite a rather comprehensive understanding of the role of bacteria associated with rice in the solubilization of silicate minerals (17, 18). New insights into the complexities of As absorption, dispersion, and the potential impact of Si highlight the importance of this characteristic. The dual influence of Si on As accumulation in rice may be amplified by As-resistant SSB, according to recent results. Implementing SSB-inoculum into simple hydroponic systems reduced As uptake by rice plants. This was achieved by increasing the availability of Si and encouraging root-based competition between As and Si for aquaporin transporters (19). Research conducted by Bist et al. (20) concluded that the silicate-solubilizing *Bacillus amyloliquefaciens* effectively reduced As levels in rice grains.

This review paper examines the integration of SSM and Si-rich agro-wastes to evaluate their dual efficacy in mitigating As levels in the final product. It highlights the potential of SSM and Si-rich agro-wastes, either individually or in conjunction, as a cost-effective and environmentally sustainable alternative to commercially available Si fertilizers. This comprehensive review on understanding and validation of the mechanism provides a valuable insight in formulating a feasible As toxicity management strategy.

## 2 Arsenic contamination and food security

Arsenic, a toxic metalloid naturally present in the Earth's crust, has become an increasingly significant threat to agriculture due to anthropogenic sources such as the use of As pesticides, mining activities, and irrigation with As-contaminated groundwater (21). One of the most critical pathways through which As impacts human health indirectly is by altering the nutritional quality of crops (22). This degradation begins at the soil-root interface, where As disrupts nutrient uptake, mobility, and assimilation, leading to deficiencies in essential macro- and micro-nutrients in edible plant parts. This section reviews in detail how As interferes with nutrient acquisition and the resulting effects on crop nutritional profiles.

### 2.1 Arsenic speciation and its interaction with nutrient pathway into plants

Arsenic exists primarily in two inorganic forms in the soil: arsenate (As^5+^) and arsenite (As^3+^), with methylated organic forms like monomethylarsonic acid and dimethylarsinic acid found to a lesser extent (23). In aerobic soils, arsenate (As^5+^) predominates and structurally and chemically mimics phosphate (${\text{PO}}_{4}^{3-}$), allowing it to compete for absorption via phosphate transporters in root cells (PHT1 family). This phosphate pathway mimicry leads to a physiological phosphorus deficiency even in P-sufficient soils (24). Under anaerobic circumstances, such as inundated paddy fields, As^3+^ emerges as the predominant species and infiltrates plant roots via nodulin 26-like intrinsic protein aquaporin channels (25). This absorption pathway indicates that As directly disrupts the transport and bioavailability of key nutrients, starting with phosphorus and extending to others via various indirect and regulatory processes (Table 1).

**Table 1:** Interaction of arsenic with nutrients and its implication on plant and human health.

**Nutrient interaction**	**Mechanism of As interaction with the nutrient**	**Implication on plant health**	**Implication on human health**	**References**
Nitrogen (N)	Arsenic suppresses nitrate transporters (notably NRT1.1 and NRT2.1 in cereals), decreases nitrate reductase and nitrite reductase activities by altering their gene expression and promoting reactive oxygen species (ROS) accumulation and interferes with ammonium incorporation into amino acids. Furthermore, As interferes with the incorporation of ammonium into amino acids via glutamine synthetase and glutamate synthase, leading to reduced pools of glutamine and glutamate—precursors for the biosynthesis of all other amino acids.	Reduced concentrations of total nitrogen, free amino acids, and protein in consumable tissues, impaired amino acid and protein biosynthesis.	Populations consuming these crops may experience reduced dietary protein intake leading to protein malnutrition, impaired growth, weakened immunity, and lowered nutritional status.	(124, 255–257)
Phosphorus (P)	In aerobic soils, arsenate (As^5+^) predominates and structurally and chemically mimics phosphate (${\text{PO}}_{4}^{3}$^−^), and competes for phosphate transporters (PHT1 family); disrupts ATP formation by substituting phosphate, creating unstable ADP-As intermediates, which decompose rapidly and dissipate cellular energy.	Impaired cellular energy metabolism, reduced active nutrient transport and metabolic activity.	Low phosphorus in foods can increase risks of bone and dental problems, poor energy metabolism, and general weakness, particularly among groups like children, pregnant women, and those with limited dietary diversity	(43)
Iron (Fe)	Arsenic downregulates iron transporter genes (IRT1, FRO2), causes oxidative stress that mobilizes iron, depleting Fe in edible parts.	As induced chlorosis in fully developed young leaves, lower iron content in edible parts (such as grains, fruits, and vegetables), directly reducing their nutritional value.	Iron-deficiency anemia (fatigue, weakened immunity, developmental and cognitive problems in children), greater susceptibility to arsenic toxicity, which impacts the skin, cardiovascular system, neurological function, and increases cancer risk	(258)
Zinc (Zn)	Zn uptake is inhibited through both competitive interactions at root uptake sites and indirect effects on membrane permeability. Zinc deficiency due to As has been associated with reduced activity of carbonic anhydrase and superoxide dismutase enzymes essential for crop health and nutritional density	Lower Zn concentration in straw, roots, grains; increased ROS, DNA modification, reduced growth hormone efficiency like auxins, gibberellins, and carotenoids.	Frequent illness or infection, slow wound healing, reduced DNA synthesis and neurotransmission, hair loss, skin rashes, white spots on nails	(259)
Manganese (Mn)	As interferes with Mn acquisition by disrupting Mn transporter expression and root oxidation capacity, which is essential for converting Mn^+2^ into absorbable forms	Damaged chloroplast structure, lowers chlorophyll content, reduces net photosynthesis, and decreases soluble sugar concentrations	Reduced fertility, impaired bone development, and metabolic disturbances, neurodegenerative disorder	(260)
Calcium (Ca)	Specific Ca^2^^+^ signals are generally detected by various Ca^2^^+^ sensors such as CALCIUM-DEPENDENT PROTEIN KINASES (CPKs), CALMODULIN (CaM), CALCINEURIN B-LIKE PROTEINS (CBLs), CALMODULIN-LIKE PROTEINS, and their interacting kinases, called CBL INTERACTING PROTEIN KINASES (CIPKs). These sensors then translate the signals into metabolic and transcriptional responses. In response to arsenic stress, the differential expression of CaMs indicates a potential role for Ca^2^^+^-dependent signaling in the arsenic tolerance mechanisms of plants. In this context, Calcium-Dependent Protein Kinases (CPKs) are key regulatory proteins that typically play a role in decoding Ca^2^^+^ signals triggered by As stress.	As stress causes cytosolic acidification, disrupting calcium signaling pathways and causing cellular leakage of Ca^2+^ ions which ultimately reduces cell wall integrity and cell membrane stability	Weak, brittle bones with risk of osteopenia and osteoporosis; muscle cramps, spasms, twitching; dental issues like enamel weakening; potential cardiovascular effects.	(44, 261)
Magnesium (Mg)	As reduces Mg availability through altered transporter function and membrane fluidity. Although docking interaction studies between 60CE protein with both Mg^2+^ and As^3+^ showed a better link with As^3+^ via hydrogen bond, it can damage the plant more effectively with Mg deficiency.	Reduced photosynthetic efficiency and nutritional development	Abnormal heart rhythm (arrhythmia), palpitations, and increased risk of cardiac arrest, obesity, insulin resistance, metabolic syndrome, and type 2 diabetes.	(26)

The competitive interaction between arsenic and essential nutrients—especially phosphorus, nitrogen, and iron—not only hampers plant health and productivity but also diminishes the nutritional quality of food crops. These interactions are critical in arsenic-exposed regions, where targeted nutrient management could mitigate arsenic toxicity and improve food safety.

### 2.2 Disruption of root architecture

**Figure 1:**
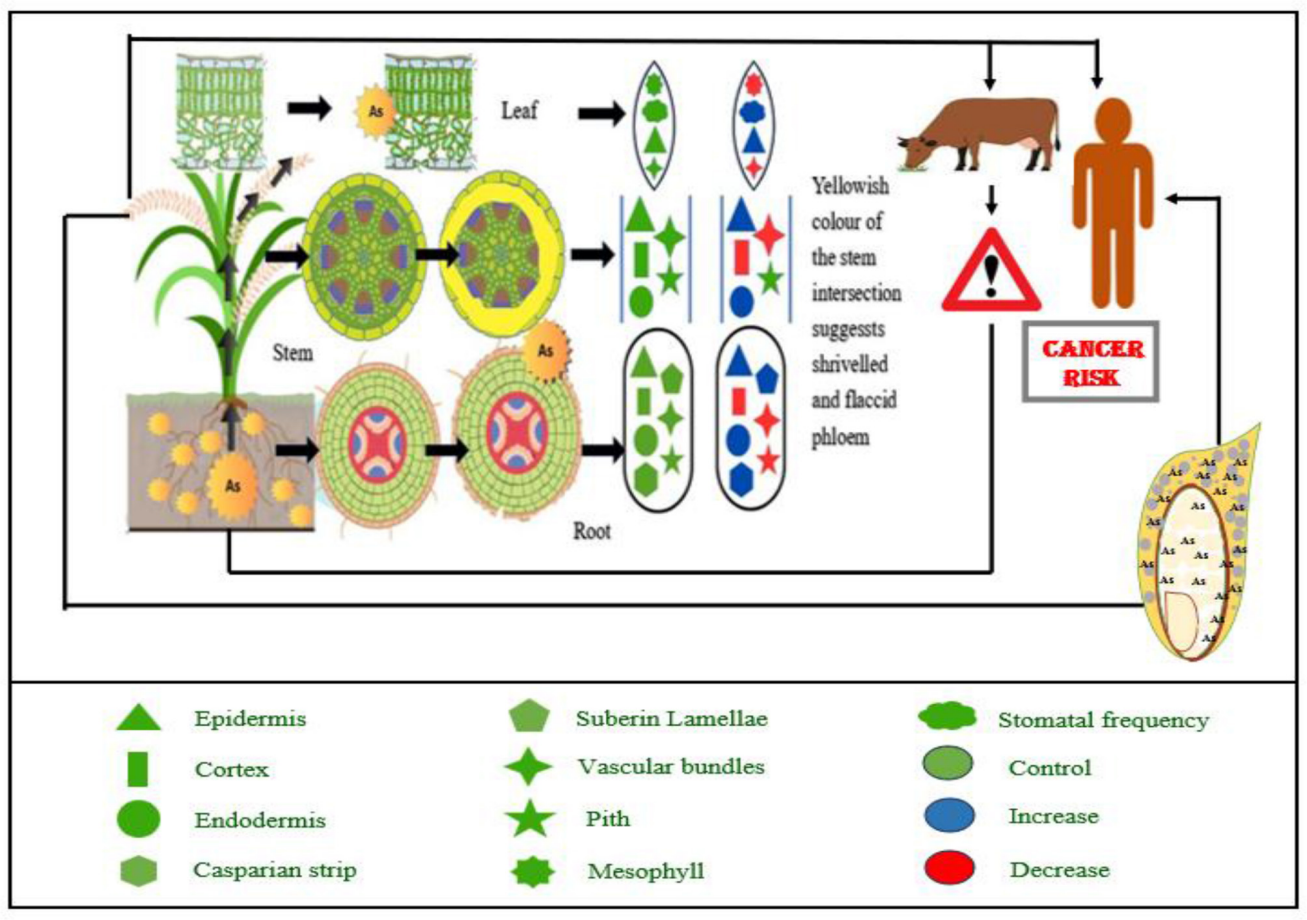
Arsenic contamination impacts food security and increases cancer risk for humans and animals by altering nutrient mobilization from soil to plants and affecting the structural organization of various plant parts. Abbreviation: As, Arsenic.

Roots are the initial organs that interact with metals and metalloids in the soil; hence, various morphological modifications of root tissues may be anticipated (Figure 1). As toxicity causes morphological changes in root systems, including reduced root length, branching, and surface area. These alterations limit the physical capacity of the root to explore soil nutrients, thereby compounding the problem of nutrient deficiency (26). Water lettuce (*Pistia stratoides*) exhibited root loss with exposure to As (27). Talukdar (28) observed a threefold and two and a half-fold decrease in root length and root dry weight, respectively, in seedlings of *Phaseolus vulgaris*. The application of As led to a notable brown discoloration of the roots, accompanied by a reduction in the development of lateral roots. The presence of As at a concentration of 2 mg L^−1^ led to the total eradication of lateral roots, leaving merely a few lateral root primordia in the cortex (29). The number of lateral roots decreased, becoming concentrated in the basal region of the roots, alongside a darkening of the roots in soybean (*Glycine max*) plants subjected to As treatment (30).

Although the root apical meristem, safeguarded by the root cap, is pivotal in influencing subsequent root growth, anatomy, morphology, and functionality (31, 32), it is the initial segment of the root that directly encounters toxic soil conditions and is consequently vulnerable to As exposure. Exposed roots often preserved the functioning of the root apical meristem concerning cellular division. The genotoxic effects of As on onion root growth were described by Gupta et al. (33). This was demonstrated by the increased frequency of micronuclei inside the intermediate phase of root meristem cells. There have been cases when arsenic's negative impacts on tap root lateral root primordia's growth and development have led to an increase in their activation along the tap root axis, which in turn has changed the root morphology. Arsenic and cadmium, according to Ronzan et al. (34), both facilitated the growth of lateral roots, which were associated with altered and weakened meristem organization. In addition, the uneven creation of the quiescent center and aberrant cell divisions in the root apical meristem prevented the emergence of several lateral root primordia from the tap root. These changes may subsequently lead to various anatomical alterations in older tissues (35).

The rhizodermis is the first root tissue affected by arsenic (As) contamination, disrupting water and nutrient uptake (35). Arsenic alters root hair development (36), often reducing or eliminating root hairs in species like *Phaseolus aureus* (37) and *P. vulgaris* (28), while *Pteris vittata*, a known As hyperaccumulator, shows minimal morphological changes (38). Cortical tissues—exo-, meso-, and endodermis—exhibit significant damage under As exposure (35), including cell disintegration, reduced parenchyma thickness (30), and dark deposit accumulation, as observed in *Glycine max* and *Cajanus cajan* (29). Structural changes also affect the central cylinder and vascular tissues. *Brassica juncea* showed increased cylinder diameter, while *B. oleracea* showed a decrease (39). As toxicity caused xylem deformation and vascular tissue destruction in *P. vulgaris* and *C. cajan*. Notably, dark deposits in vascular tissues were more pronounced under As(III) than As(V) (30). These findings highlight the species-specific morphological responses and the detrimental impact of arsenic on root structure and function (Figure 1).

### 2.3 Changes in stem tissue anatomy

The stem is the part of the plant organ that links the roots with the primary photosynthetic organs, which are the leaves. One of the primary roles of the stem is to support and transport nutrients to leaves and blossoms. Metals and metalloids are conveyed to aerial organs via vascular tissues; hence, the vasculature and its environs are often the locus of notable morphological modifications within stem tissues (35). Sclerenchymatous cells next to the phloem become desiccated and limp after As exposure, which impedes water transport and causes abnormalities in the phloem cells of the stem (40). The introduction of As led to the formation of crystals and druses within the epidermal layer, vascular bundles, cortex, and pith region of the stem (28).

### 2.4 Modifications in leaf tissue anatomy

The leaf functions as the central organ of photosynthesis, an essential process that generates the energy required to maintain physiological functions throughout all plant tissues. The predominant approach employed by many plants involves limiting the absorption and movement of heavy metals and metalloids to aerial structures, thus protecting photosynthetically active tissues from the adverse impacts of these toxic elements (35). Numerous findings indicate that leaf thickness has diminished as a result of the inclusion of metalloids. This was noticed as a result of the presence of As (39, 41). The narrowing of xylem channels (Figure 1) in the leaves of various plant species due to As exposure has been documented (41, 42).

### 2.5 Impact on plant metabolism

#### 2.5.1 Impact on photosynthesis

**Figure 2:**
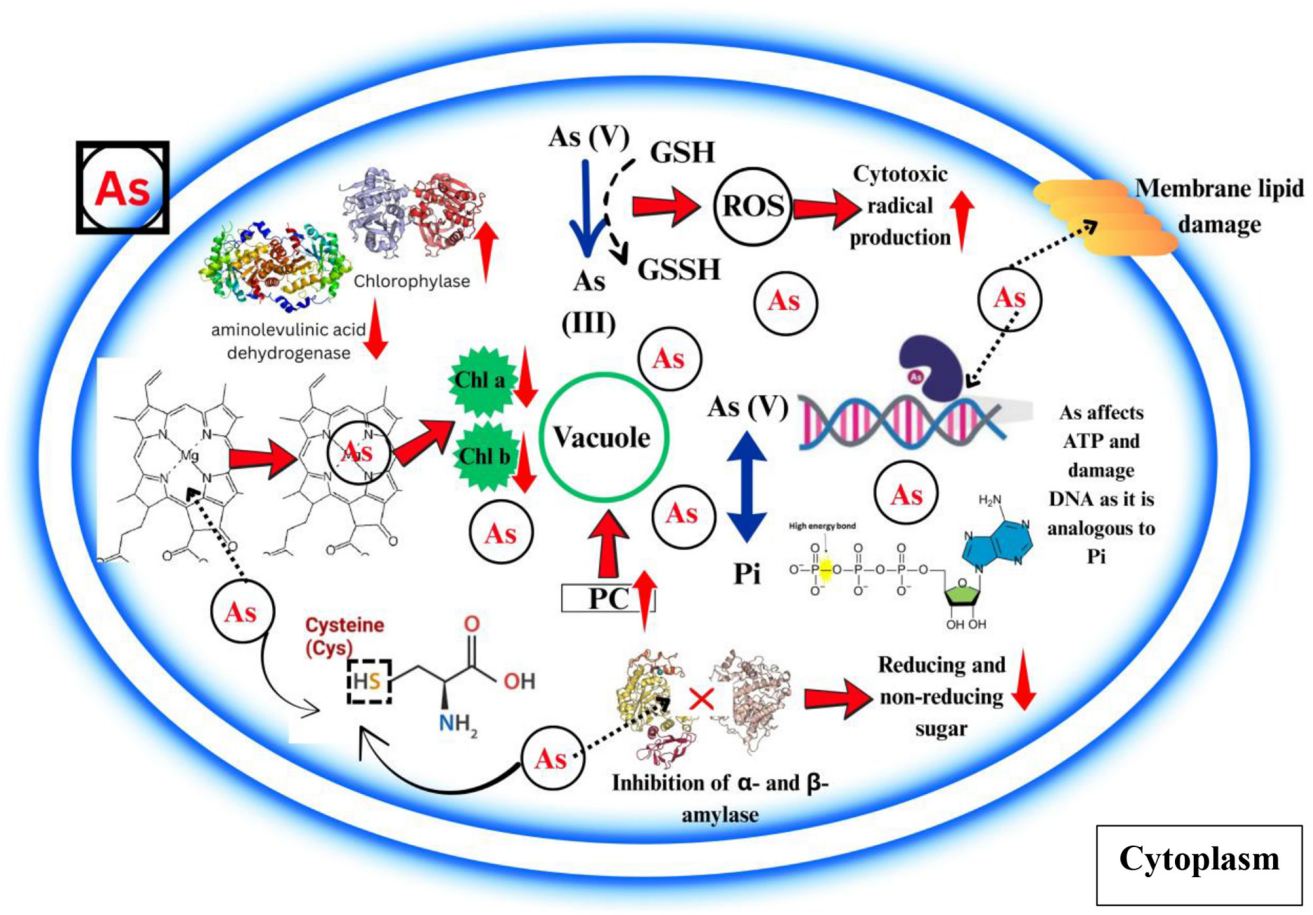
The effect of arsenic phytotoxicity on photosynthetic pigments, protein, carbohydrate and lipid molecules. It is shown how arsenic can compete with Pi in the metabolic processes that require Pi. Abbreviations: Arsenic (As), Inorganic arsenic [As (III)], Arsenite [As(III)], Arsenate [As**(V)**], Phosphorus (Pi), Reactive oxygen species (ROS), Glutathione (GSH), Oxidized glutathione (GSSH), Phytochelatin (PC), Adenosine triphosphate (ATP), Deoxyribonucleic acid (DNA).

According to various research, As accumulation greatly hinders photosynthesis process (43, 44) (Table 2; Figure 2). According to the mainstream view, the previously described inhibition is linked to ROS accumulation which is caused by As and their discrepant effect on basic photosynthetic mechanism. Kalita et al. (45) posited that, contrary to conventional understanding, oxidative stress resulted from the suppression of photosynthesis at lethal concentrations of As. Accumulation leads to a substantial decrease in chlorophyll concentration (46, 47). While As has a greater impact on chlorophyll synthesis, it has a smaller effect on the degradation of carotenoid pigments, which is linked to the reduction of chlorophyll in As-grown plants (48, 49).

**Table 2:** Effects of arsenic phytotoxicity on plant metabolism.

**Impact on plant metabolism**		**Observed effect under As stress**	**Proposed mechanism**	**References**
Photosynthetic components	Chlorophyll a (Chl a)	Decline in Chl a concentration and the inhibition or diminished availability of precursors such as d-aminolevulinic acid	Suppression of d-aminolevulinic acid dehydrogenase activity, elevated activity of chlorophyllase, Mg^2^^+^ replacement by As(III) in tetrapyrrole ring	(262, 263)
	Chlorophyll b (Chl b)	Inhibition of activity	Oxidative damage, reduction in Chl a content	(48)
	Carotenoids	Variable: decrease or increase	Inhibition of precursor synthesis/ROS-induced non-enzymatic antioxidant response	(264–266)
	PS II	Reaction center inactivation, lowered plastoquinone reduction, decreased OJIP kinetics including Fv/F0 values	Oxidative damage to thylakoid proteins and D1 protein turnover, blocking effect on the donor end of PS II	(48, 267–269)
	Dark reaction	Minimal impact	Primary target is light reaction components	(270)
Protein metabolism		Reduction in total protein content, enzyme inhibition, protein carbonylation	As binding to sulfhydryl groups; inhibition of nitrate/nitrite reductases; ROS-induced oxidation of amino acid residues	(53, 59)
Lipid metabolism		Lipid peroxidation, membrane damage, altered lipid biosynthesis gene expression	ROS-induced peroxidation; altered expression of lipid synthesis genes; cytotoxic radical production	(53, 56)
Carbohydrate metabolism		Decrease in reducing and non-reducing sugars; inhibition of starch-degrading enzymes	Suppression of sucrose synthesis; inhibition of starch phosphorylase, α- and β-amylase; altered hexose monophosphate pathway	(50, 51)

#### 2.5.2 Impact on protein, lipid, and carbohydrate metabolism

The presence of As adversely impacts the metabolic processes (Table 2; Figure 2) of vital carbohydrates, such as sugars and starches. The incorporation of As in *Oryza sativa* led to a decrease in both reducing (hexoses) and non-reducing (sucrose) sugars in the shoots (50), suggesting a suppression of sucrose synthesis in comparison to hexose monophosphate. Its phytotoxicity was enhanced because it significantly suppressed the functions of enzymes that break down starch, namely starch phosphorylase and α- and β-amylase. On the other hand, when stress was applied to *Oryza sativa* and *Phaseolus aureus* seedlings, it increased starch phosphorylase activity, leading to higher levels of soluble sugars (51).

The stress induced by As leads to lipid oxidation, a process considered significantly harmful to plants. Cellular electrolyte leakage and membrane degradation were significantly enhanced in several plant species that were subjected to As stress (52–54). According to Clemens and Ma (55), the peroxidation of lipid molecules within cellular and organelle membranes is influenced by the elevated level of ROS caused by As. In the end, the cytotoxic radicals that are mediated by lipids damage the functionality of cells or tissues. It has been discovered that As exposure alters the mechanism for lipid synthesis. Significant changes in the expression of 59 genes associated with lipid formation were seen in a comparative transcriptome analysis of rice following exposure to As(III) treatment (56). Despite evidence that As affects genes involved in lipid formation, studies elucidating how As affects plant lipid levels are scarce. The strong binding of inorganic As compounds to sulfhydryl groups in proteins causes damage to plant cell membranes and eventual cell death, significantly interfering with plant metabolism. The total protein content in plants is reduced when As is present (53). The external introduction of As impeded the activity of nitrate and nitrite reductase, enzymes integral to the reduction of protein concentrations in plants. The disintegration of proteins into individual amino acids is primarily facilitated by proteases and peptidases. A reduction in exposure leads to diminished protease levels, subsequently hindering the growth and development of plants (57). The trivalent form of As can bind directly to the sulfhydryl groups of proteins and obstruct several biological pathways; in contrast, the pentavalent form acts as a phosphate analog and disrupts phosphorylation activities (58). According to Fedorova et al. (59), proteins undergo carbonylation changes due to an overabundance of ROS produced by As stress. Proteins incorporate carbonyl (C=O) groups either directly or indirectly via interactions with reactive carbonyl species or the oxidation of certain amino acids (60). When their side chains are oxidized, some amino acids that are known to be proteinogenic—including arginine, histidine, lysine, proline, threonine, and tryptophan—are able to form carbonyl groups. Biomolecular impairment, increased toxicity, and the induction of apoptotic cell death are caused by the increased presence of carbonyl compounds, which are a result of reactive carbonylated species and their interactions with nucleophilic substrates (61).

### 2.6 Impact on soil microbial activity

Arsenic (As) contamination in soils presents a substantial risk to the ecological viability of agroecosystems by adversely affecting microbial populations, enzymatic activity, and nutrient cycling. These biological disruptions impair soil health and diminish plant productivity, thereby jeopardizing long-term food and nutritional security. Li et al. (62) documented a significant alteration in microbial community composition due to As stress, characterized by a rise in Gemmatimonadota and a decrease in Bacteroidota and Nitrospirota. In the arsenic-contaminated soils of the Bengal Delta Plain, significant alteration of microbial species including Alpha-, Beta-, and Gamma-proteobacteria, Actinobacteria, and Acidobacteria was reported (63). These groupings, functionally associated with soil nutrients such as nitrogen, potassium, phosphate, and iron, exhibited a negative correlation with increasing arsenic levels. Evaluations of microbial activity using basal respiration, substrate-induced respiration (SIR), and fluorescein diacetate (FDA) hydrolysis demonstrate persistent declines in arsenic-contaminated soils. Ghosh et al. (64) documented an elevation in the microbial metabolic quotient (qCO_2_), signifying increased respiratory stress in relation to microbial biomass carbon. The decline in FDA hydrolysis was ascribed to the inhibited production of hydrolyzing enzymes (protease, lipase, esterase) and diminished fluorescein absorption and release in microbial cells (65). Soil enzyme activities—specifically β-glucosidase, arylsulfatase, urease, and both acid and alkaline phosphatase—diminish markedly with elevated labile As concentrations. Bhattacharyya et al. (66) exhibited significant negative associations between these enzymatic activities and exchangeable or water-soluble arsenic components. The activity of alkaline phosphatase is notably sensitive because of the structural resemblance between As(V) and phosphate, resulting in competitive inhibition (67). Nonetheless, urease exhibited merely a 33–38% decrease, suggesting a diminished direct reliance on arsenic concentrations (68). Environmental variables additionally influence these consequences. Enzyme activities were significantly inhibited under anaerobic circumstances, like those in paddy fields, compared to aerobic soils, owing to microbial sensitivity to oxygen (69). Anaerobic respiration with low molecular weight organic acids (e.g., acetate, formate) facilitates arsenic desorption and impairs enzymatic activity (70). The microbial reduction of iron oxyhydroxides increases arsenic solubility at low redox potential, intensifying enzyme inhibition, particularly for glucosidase (68).

Soil microbial communities demonstrate differing tolerances to arsenic species. Guan et al. (71) discovered that As(III)-tolerant bacteria and actinomycetes are present in lesser quantities than their As(V)-tolerant equivalents, but fungus exhibited comparable resistance to both As(III) and As(V), indicating superior fungal resilience. Despite these detrimental impacts, certain microbes possess arsenic-detoxifying abilities, such as As(V) reduction, As(III) oxidation, methylation, or sequestration in biomass (Section 3.2). These groups can reduce arsenic mobility and bioavailability, indirectly reducing plant uptake of As. Harnessing such microbial processes—either naturally occurring or through bioaugmentation—can complement other remediation strategies.

As both microbial processes and soil amendments can influence arsenic speciation and mobility, integrating microbial remediation with silicon (Si) supplementation offers a synergistic approach. While Si reduces arsenic bioavailability through adsorption, precipitation, and competition with phosphate uptake, beneficial microbes can further enhance this effect by immobilizing or transforming arsenic into less bioavailable forms. Together, they form a dual strategy for mitigating As bioaccumulation in crops.

## 3 Role of Si in mitigating As bioaccumulation

Silicon, although not essential for plant growth, confers numerous physiological benefits and interact in the soil environment through several mechanisms, primarily affecting bioavailability of As and its uptake by plants.

### 3.1 Si-mediated iron plaque–As interaction in plants

**Figure 3:**
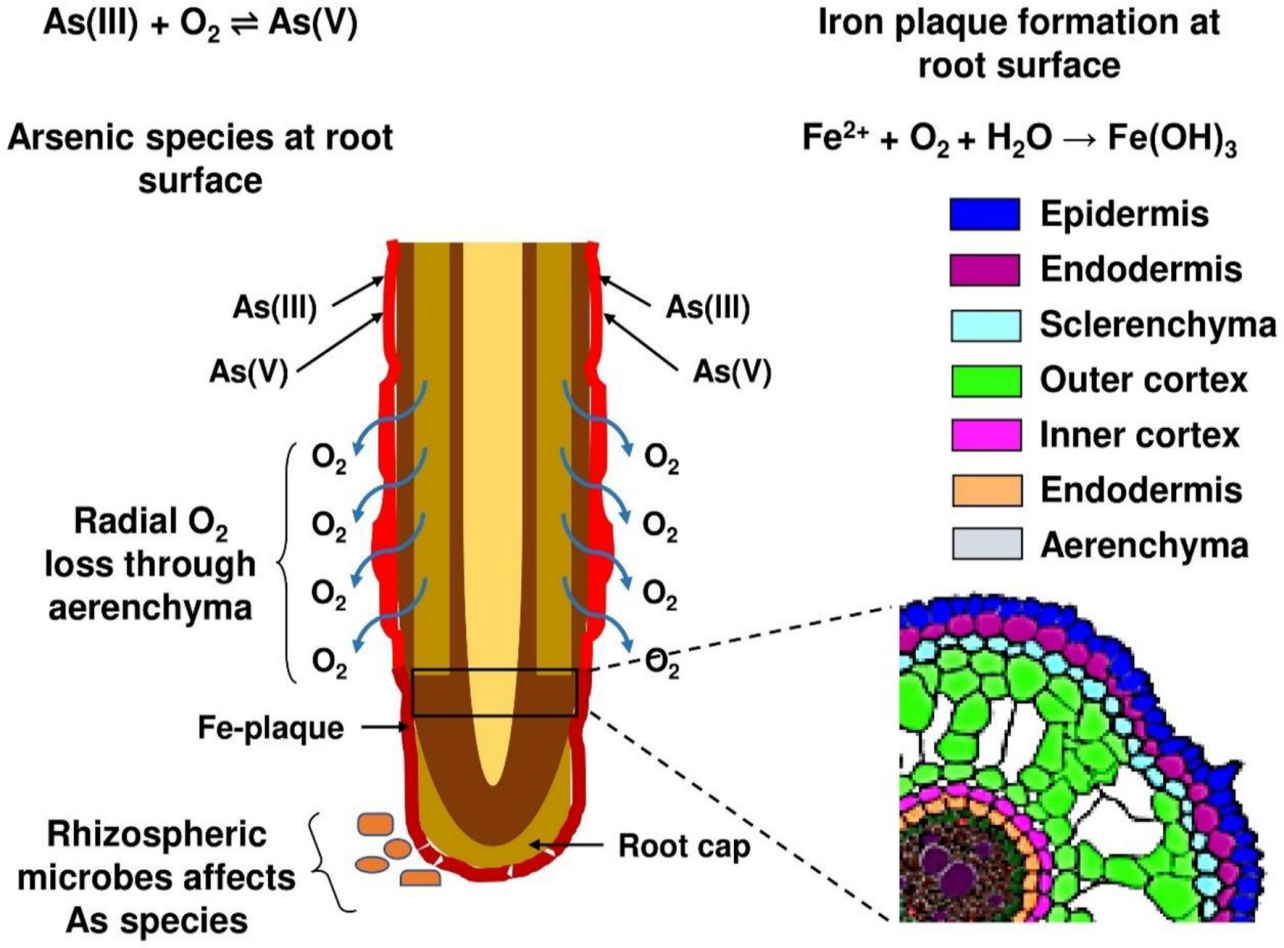
The influence of Fe plaque formation on rice root surface on As availability.

**Figure 4:**
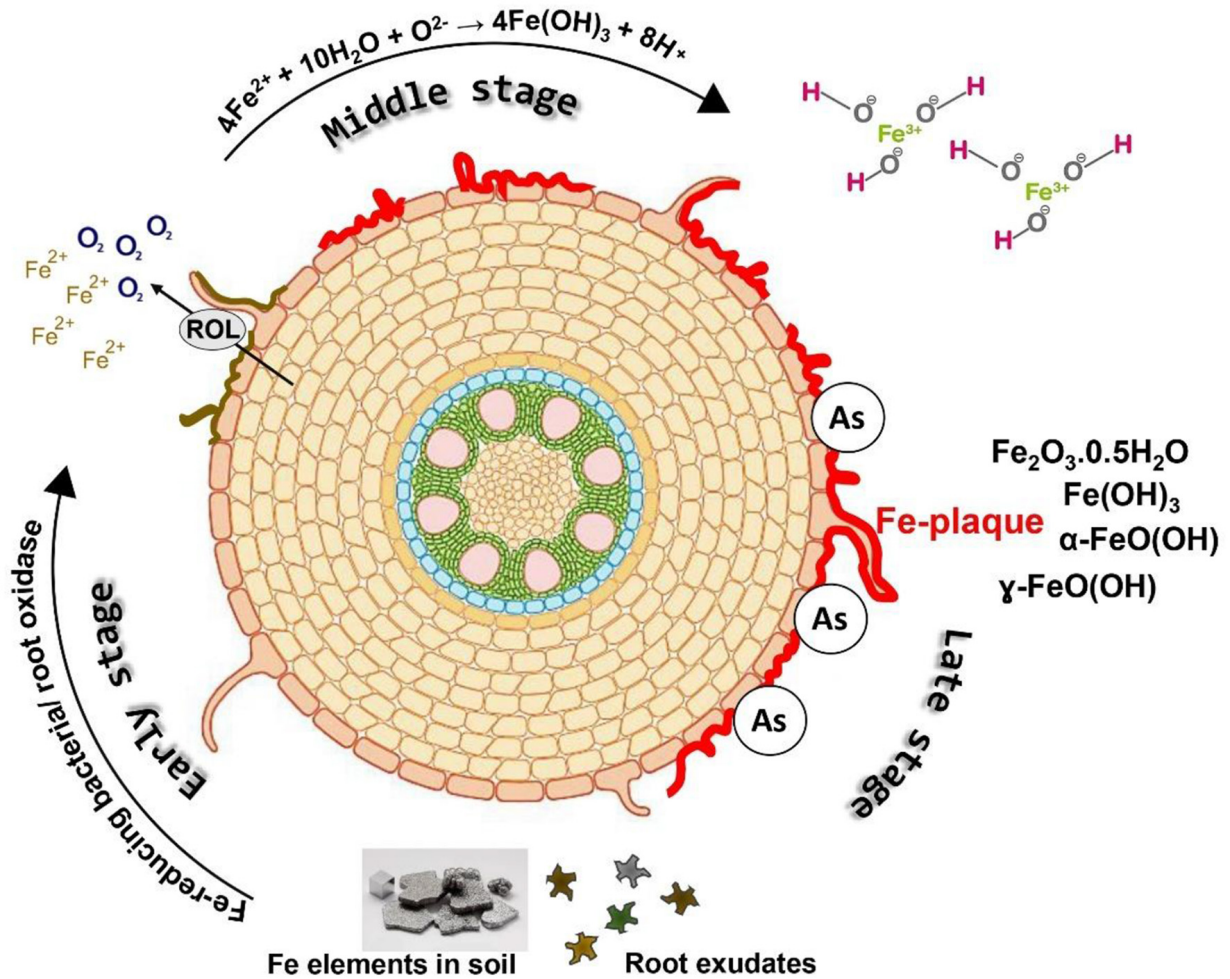
The formation process of Fe plaque (IP) through various oxidation-reduction processes occurring outside the root.

The interaction between Si, iron (Fe) plaques, and As at the root–soil interface is a critical process influencing As uptake and toxicity in wetland crops, especially rice (Figure 3). Rice cultivated in inundated soil conditions, along with other aquatic flora, develops a Fe plaque on the root surfaces as a result of pronounced redox gradients from the roots to the reduced bulk soil. The diminished bulk soil is defined by the reductive dissolution of iron oxide minerals, leading to elevated Fe(II) concentrations in the soil solution (25, 72). Oxygen escaping from the expanded gas cavities of aerenchyma tissue into the rhizosphere, known as radial oxygen loss, significantly influences the redox chemistry in close proximity to the root (73, 74). The oxic rhizosphere facilitates the fast oxidation of porewater Fe(II) to insoluble Fe(III) precipitates on the exterior of roots, predominantly at root tips and lateral root junctions (75, 76). Figure 4 depicts the sequential formation of Fe plaque in distinct stages. This Fe plaque is predominantly made up of the Fe oxyhydroxides ferrihydrite [Fe(OH)_3_·nH_2_O], lepidocrocite (γ-FeOOH), and goethite (α-FeOOH) (77, 78). The elevated zero charge potential of FeOx (>7) facilitates the formation of robust inner-sphere adsorption complexes with various anions and promotes adsorption at edge and corner sites (79). Porewater containing arsenate [As(V), H_3_AsO_4_] and arsenite [As(III), H_3_AsO_3_] exhibits significant monodentate or bidentate complexation with iron plaque (80). Ferrihydrite is a highly reactive mineral that initially predominates in the rhizosphere but can subsequently convert into the more crystalline forms of lepidocrocite and goethite over time. Anions adhere to the edge and corner sites of FeOx, with ferrihydrite exhibiting a greater abundance of the more robust edge sites compared to the other two (81).


$$\begin{array}{l} \equiv \text{FeOH}+{\text{H}}_{2}\text{As}{\text{O}}_{4}^{-}\to {\left(\equiv \text{FeO}\right)}_{2}\text{As}{\text{O}}_{2}^{-}+{2\text{H}}_{2}\text{O} \end{array}$$

Under dynamic redox conditions, As(V) or As(III) can be immobilized through coprecipitation with Fe oxides during Fe(II) oxidation and Fe(III) hydrolysis (82). Arsenic becomes structurally incorporated within the Fe oxide matrix as it forms:


$$\begin{array}{r} \text{F}{\text{e}}^{2+}+\frac{1}{4}{\text{O}}_{2}+\frac{5}{2}{\text{H}}_{2}\text{O}\to {\text{Fe}\left(\text{OH}\right)}_{3}\left(\text{s}\right)+2{\text{H}}^{+}\\ {\text{H}}_{3}\text{As}{\text{O}}_{4}+{\text{Fe}\left(\text{OH}\right)}_{3}\left(\text{s}\right)\to \text{Fe}-\text{As coprecipitate } \end{array}$$

Si can be adsorbed onto or co-precipitated with Fe oxides during plaque formation. The incorporation of Si into Fe oxides interferes with their structural ordering due to steric hindrance and disruption of Fe–O–Fe bonding, which alters nucleation and crystal growth kinetics (83). Si incorporation has a retarding effect on Fe oxide crystallization by binding to surface hydroxyl groups and blocking reactive sites necessary for phase transformation. This results in the stabilization of poorly crystalline phases like ferrihydrite over more crystalline forms like goethite or hematite, both under pure mineral systems and in rice root experiments (78, 84). The resulting plaques exhibit higher specific surface areas, greater sorption capacities for metals like arsenic (As), and altered redox reactivity. Hence, elevated concentrations of Si in porewater may enhance the retention of As by promoting the formation of ferrihydrite-dominated Fe plaques (85). Moreover, Si nutrition benefits rice plants growth and improves oxygen secretion ability of the roots, maintaining an oxic microenvironment for plaque formation strength and silicate anions compete with arsenite for sorption sites, thereby increasing As mobility in the (86). But under Si-rich flooded condition, reduction of arsenate to arsenite decreases its adsorption rhizosphere (85, 87).

Gu et al. (88) observed that Fe content in amorphous fraction of plaque (AIP) was higher than the crystalline fraction (CIP) and further increased (40.8–205.8% in AIP and 2.9–187.9% in CIP) after supplying Si-rich rice husk ash (RHA). Compared with non-RHA addition, the As contents in the AIP and CIP increased by 22.4–235.6% and 51.5%, respectively, with HA supplication at low-concentration single As stress. The application of HA reduced As contents in the shoots and roots by 31.9–42.8% and 9.9–17.9%, respectively at single As stress. Jiang et al. (89) reported an increased Fe and As content in plaque by 9.4–53.7% and 28.0–33.1%, respectively, after application of 0.5–2.0% RHA. Compared to no-RHA treatments, 0.5–2.0% RHA treatments significantly reduced the As contents in stem, leaves and roots by 50.0–78.8%, 16.8–82.8% and 14.9–38.1%, respectively. 2.0% RHA application decreased inorganic As content in brown rice by 30.8% compared to no-RHA treatment. Khanam et al. (10) showed co-application rice straw compost (RSC) and SSB resulted in the maximum Fe plaque formation with a concentration of 3,140 mg kg^−1^, followed by the sole RSC (2,911 mg kg^−1^), which were significantly higher than the control (2,321 mg kg^−1^). Leksungnoen et al. (90) found that Si-rich RHA (0.64% w/w) almost doubled that As concentration in Fe plaque compared to untreated plots and plaque As was higher that compared to RHB.

### 3.2 Microbe mediated immobilization of As

**Figure 5:**
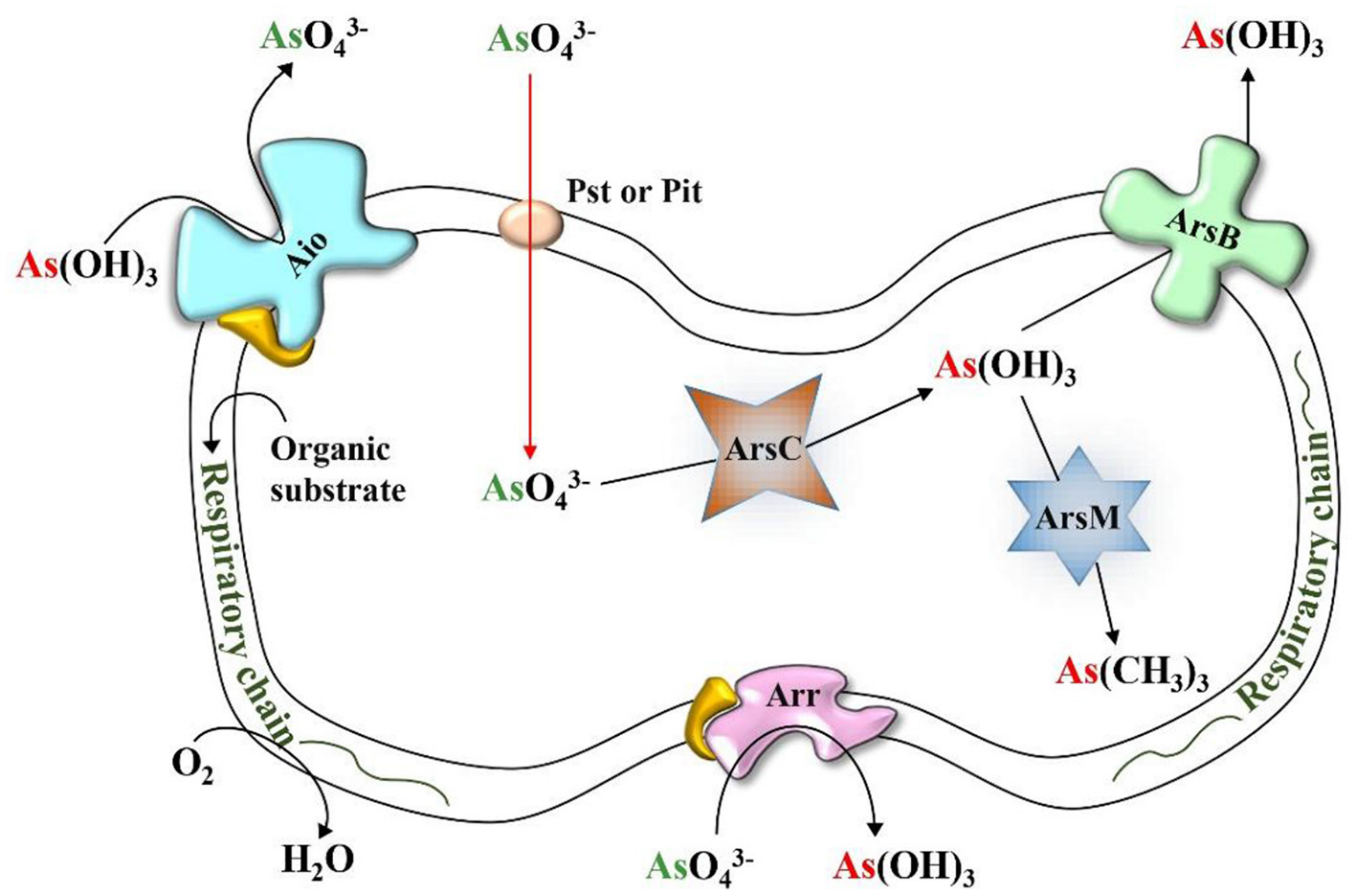
Cellular locations and functioning of microbial enzymes involved in As immobilization.

A variety of bacteria associated with the rice rhizosphere can play a role in the biotransformation of As (As) by oxidizing As(III), reducing As(V), methylating As(III), and respiring As(V) (91). Microorganisms containing As functional genes, including arsenite oxidase, arsenate reductase, respiratory arsenate reductase and arsenite methyltransferase play a key role in regulating the speciation and mobility of As in paddy soil as shown in Figure 5 (92). The oxidation and methylation of As(III) are recognized as natural detoxification pathways of the As biotransformation cycle in the paddy rice system (91, 93).

The archetypal *aio* system, the *aioBA* operon, was first identified and completely sequenced from the β-proteobacteria *Herminiimonas arsenicoxydans*. It encodes arsenite oxidase (Aio), comprising two subunits: AioA, the large molybdopterin-containing catalytic unit, and AioB, a small Rieske [2Fe-2S] cluster protein (94). Aio catalyzes the oxidation of As(III) to As(V) through four sequential electron transfer steps (95). Stopped-flow spectroscopy and isothermal titration calorimetry revealed that As(III) binds near a funnel-shaped cavity of AioA, where polar residues coordinate it via the molybdopterin cofactor. The bound As(III) donates electrons to the Mo(VI) center, reducing it to Mo(IV) while being oxidized to As(V) at rates exceeding 4,000 s^−1^. Electrons are then rapidly transferred from Mo to the Rieske centers. The final, rate-limiting step involves electron transfer from the AioB Rieske cluster to the terminal electron acceptor, cytochrome c, completing the catalytic cycle (96).

Microbial methylation, or biomethylation, refers to the biological transformation of metals and metalloids into volatile and nonvolatile methylated compounds with the help of methyltransferase enzyme (97). First identified in fungi, this process is crucial for As detoxification and its environmental cycling. The *arsM* gene enables microbes to methylate and resist As toxicity (98). The most widely accepted pathway, proposed by Challenger et al. (99, 100), involves initial reduction of As(V) to As(III), followed by two successive enzyme-mediated reductions. Each reduced As(III) intermediate undergoes methylation, ultimately forming trimethylarsine (Figure 4). S-adenosylmethionine (SAM) serves as the primary methyl group donor, though some anaerobic bacteria may use methylcobalamin (97).

Microorganisms reduce As(V) via two distinct pathways: the first involves cytoplasmic arsenate reductases encoded by the *ars* operon, and the second utilizes dissimilatory or respiratory arsenate reduction mediated by the *arr* gene cluster (Figure 4) (101). Serendipitously, *ars* genes were originally discovered during studies on antibiotic resistance in *Staphylococcus aureus*, not through direct investigation of arsenic resistance. Each gene in the *ars* operon contributes uniquely to arsenic detoxification: *arsR* encodes a transcriptional repressor of the SmtB/ArsR family (102); *arsA* encodes an ATPase that, along with ArsB, forms an ATP-dependent As(III) efflux pump (103); *arsD* encodes a metallochaperone that binds As(III) and transfers it to the ArsAB pump (104); and *arsC* encodes a cytoplasmic arsenate reductase, converting As(V) to As(III) (105). Alternatively, the *arr* operon, first characterized in *Shewanella* sp. ANA-3, encodes respiratory arsenate reductase ArrAB. The ArrA subunit, a large protein containing a bis-molybdopterin guanine dinucleotide cofactor and a [4Fe−4S] cluster, catalyzes As(V) reduction. ArrB, the smaller subunit, harbors four [4Fe−4S] clusters that facilitate electron transfer. This system enables anaerobic respiration using As(V) as a terminal electron acceptor, contributing to arsenic cycling under anoxic conditions (106).

The abundance of these As functional genes is generally dependent on the bacterial community structure that can be evaluated based on the diversity of 16S *rRNA* genes. The incorporation of Si-rich agro-wastes amendments, such as rice straw and rice husk, enhances soil organic matter and reduces soil redox potential, thereby directly affecting the soil microbiota (107). Porewater inorganic As(III) levels can be increased by elevated organic matter in two ways: first, by enriching an anaerobic microbial community that may be pivotal in As methylation; and second, by increasing the activity of Fe-reducers and As-reducers. Amendments high in Si had different effects on the total microbial population and the specific group of microbes that methylated As (108). Elevated calcium from calcium silicate treatments enhanced carbon storage in the first year, leading to carbon release in the second year, which may have influenced the distribution of both 16S rRNA and arsM genes. Modifications to the arsM community composition may have been impacted by reduced porewater redox potentials caused by rice husk amendment. In their study, Das et al. (109) found that indica rice grains had a 28% reduction in As and in Japonica rice grains a 30% reduction after being treated with slag-based Si. Additionally, the application of this Si increased the number of bacteria that were As-resistant and arsenite-oxidizing, which helped the soil naturally attenuate the As. Herath et al. (92) examined three different types of modified rice husk biochar (RHBC): unmodified RHBC, Si-modified RHBC, and nano-montmorillonite clay modified RHBC. The results showed that Si-RHBC significantly raised the number of bacteria (16S rRNA gene) and doubled the number of aioA gene copies compared to RHBC, which was already 25% higher than the control. The arrA, arsC, and arsM gene copy numbers were somewhat upregulated with Si-RHBC, but this effect did not reach statistical significance. The results suggest that bacteria in paddy soil that are connected with the aioA gene may help with the anaerobic oxidation of As(III) to As(V). Soil treated with Si-RHBC also showed a marked decrease in the relative abundance of Fe-reducing bacteria, particularly Bacillus and Geobacter. This suggests that the decreased abundance of these bacteria in paddy soil leads to a drop in the dissolution of As(III) from iron oxide minerals. In their study, Gao et al. (86) showed that reducing bacteria, Anaeromyxobacter and Geobacteraceae, and levels of As(III) and Fe in the rhizoplane were significantly increased by adding Si. This, in turn, inhibited the uptake of As(III) into roots.

### 3.3 Competition for transport pathways in plants

Si is an essential element in the soil and crust of the earth, but only 0.1 to 0.6% is soluble (110). Plants absorb Si as ionized Si(OH)_3_O and silicic acid. Si and As, specifically arsenite, As(III), exhibit striking chemical similarities under soil solution conditions. Both exist as uncharged molecules at typical pH ranges: H_4_SiO_4_ and H_3_AsO_3_ (arsenous acid) (111). Due to this similarity, plants, particularly rice (*Oryza sativa*), inadvertently take up As(III) using the same transporter systems that are primarily involved in Si uptake (112). The two major transporters identified for this dual uptake mechanism are Low silicon 1 (Lsi1) and Low silicon 2 (Lsi2). Both transporters play complementary roles in Si transport, yet they differ in their structure, localization, transport mechanisms, and energy dependence.

Lsi1, a member of the NIP subfamily of aquaporins within the major intrinsic protein (MIP) superfamily, is a passive channel facilitating silicic acid influx via facilitated diffusion, characterized by ar/R selectivity filters and NPA motifs (113, 114). In contrast, Lsi2 is not an aquaporin but a secondary active efflux transporter likely driven by a proton gradient, functioning as a putative anion transporter (115, 116). Unlike Lsi1, structure of Lsi2 remains less defined, though its functional role is critical for Si translocation.

**Figure 6:**
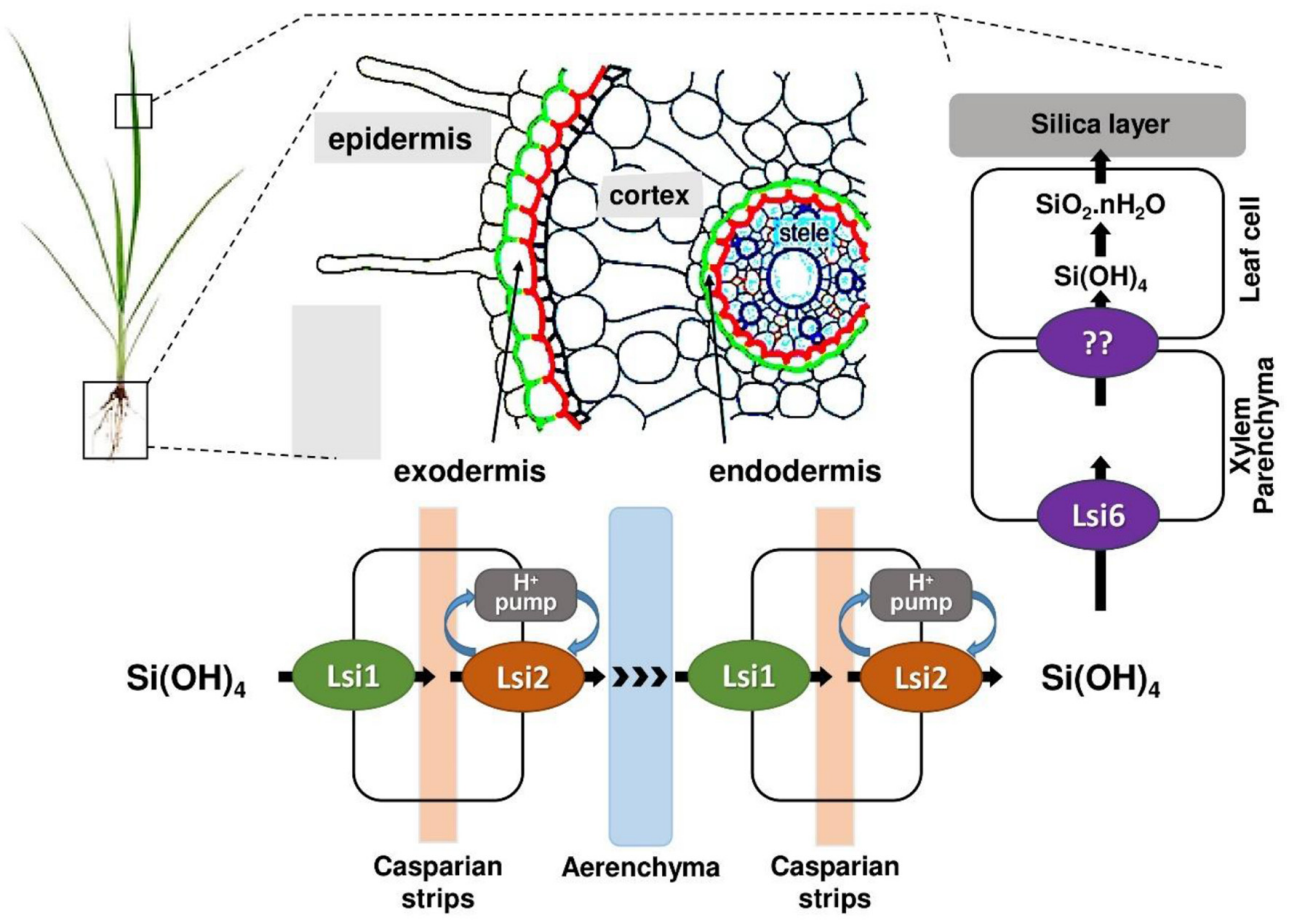
The existing Si transport model in rice roots. Lsi1 is expressed at the distal end, while Lsi2 is expressed at the proximal end.

Both Lsi1 and Lsi2 are polarly localized in the plasma membranes of root cells but on opposite sides. Lsi1 is localized on the distal (outer) side of both exodermis and endodermis cells as shown in Figure 6, facilitating the influx of silicic acid from the soil into root cortical cells (113, 117). On the other hand, Lsi2 is localized on the proximal (inner) side of the same cells, promoting the efflux of silicic acid from the root cells into the stele (Figure 6), enabling xylem loading and translocation to the shoot (115). Lsi1 operates via passive transport, relying solely on the concentration gradient of silicic acid. It does not require energy input in the form of ATP or electrochemical gradients. This aligns with its role as a bidirectional channel that can facilitate both influx and efflux depending on substrate concentration (118). Whereas, Lsi2 functions via an active transport mechanism, coupling the efflux of silicic acid with the inward movement of protons. This energy-dependent process enables Lsi2 to transport silicic acid against its concentration gradient, a necessary step to move Si from root cortical cells into the xylem (115).

Subsequent to absorption, over 95% of Si is swiftly translocated to the xylem by both Lsi2 and Lsi3 in rice. Lsi3, a homolog of Lsi2, is situated in the root pericycle cells and helps in xylem loading of Si (119). The unloading of Si from the xylem into leaf is facilitated by Lsi6, a homolog of Lsi1. Lsi6 is positioned in a polar manner on the adaxial side of the xylem parenchyma cells within the leaf sheaths and leaf blades (120, 121). Basically, Lsi6 and Lsi3 play a role in distributing silicon within the plant, including loading Si into the xylem and unloading it in specific tissues like leaf sheaths. Each plant contains specialized transporters for the uptake and accumulation of Si in various sections, such as OsLsi (Rice), TaLsi (Wheat), and ZmLsi (Maize), as indicated in Table 3.

**Table 3:** Literature survey of Si specific genes and transporters or sub families aquaporins of various plants.

**Transporter/aquaporin**	**Plant species**	**Specific genes**	**Type and expression sites**	**Functional significance**	**References**
Lsi1	*Oryza sativa*	*OsLsi1*	Influx; basal roots	Facilitates passive transport of silicic acid [Si(OH)_4_] into root cells following the concentration gradient; first step in Si uptake; mutations in Lsi1 severely reduce Si accumulation, leading to weaker stress tolerance and lower yield stability.	(113, 271)
	*Hordeum vulgare*	*HvLsi1*	Influx; basal roots		(272)
	*Triticum aesativum*	*TaLsi1*	Influx; roots		(273, 274)
	*Zea mays*	*ZmLsi1*	Influx; roots		(275, 276)
	*Sorghum bicolor*	*SbLsi1*	Influx; roots		(277)
	*Cucurbita moschata*	*CmLsi1*	Influx; roots and shoots		(114)
	*Solanum lycopersicum*	*SlLsi1*	Influx; root		(278)
	*Cucumis sativus*	*CsLsi1*	Influx; root tips		(279)
Lsi2	*Oryza sativa*	*OsLsi2*	Efflux; main and lateral roots (not in root hairs)	Actively exports Si from root cells into the apoplast toward the xylem, working in tandem with Lsi1 to achieve directional Si transport; Essential for loading Si into xylem; disruption of *Lsi2* leads to Si retention in root tissues and impaired long-distance transport.	(115, 280)
	*Hordeum vulgare*	*HvLsi2*	Efflux; basal roots		(272, 275)
	*Zea mays*	*ZmLsi2*	Efflux; basal roots		(275, 276)
	*Cucurbita moschata*	*CmLsi2*	Efflux; roots and shoots		(114)
	*Equisetum arvense*	*EaLsi2*	Efflux; roots and shoots		(281)
Lsi3	*Oryza sativa*	*OsLsi3*	Influx; Panicles	Facilitates unloading of Si into xylem transfer cells in upper nodes to ensure distribution to panicles and flag leaves; Regulates partitioning of Si to developing reproductive organs	(282)
Lsi6	*Oryza sativa*	*OsLsi6*	Influx; leaves	Mediates inter-vascular transfer and redistribution of Si within shoots, particularly toward developing tissues; Critical for optimizing Si allocation within shoots, ensuring enhanced stress resistance	(120)
	*Hordeum vulgare*	*HvLsi6*	Influx; leaf blade and sheaths		(283)
	*Zea mays*	*ZmLsi6*	Influx; leaf blade and sheaths		(275)
Aquaporins like MIP, NIP etc.	*Equisetum arvense*	*EaNIP3;1*	Influx; roots and shoots	Provide a structural and evolutionary framework for specialized channels such as Lsi1 and Lsi6, which evolved for Si transport; Key mediators of Si homeostasis, influencing plant stress adaptation, detoxification of arsenite, and efficient nutrient management.	(284)
	*Glycine max*	*GmNIP2*	Influx; roots and shoots		(285)

These transporters, primarily evolved for Si uptake, inadvertently become conduits for a toxic metalloid. This functional convergence presents a critical interface in As-contaminated environments, especially in paddy fields where anaerobic conditions favor the prevalence of As(III). The dual uptake mechanism is not merely a biochemical or physiological curiosity but a pressing agronomic challenge, as it tightly links beneficial and toxic element transport. Advances in protein modeling and transporter engineering have opened new avenues to selectively modify Lsi1 pore architecture and selectivity filters (e.g., ar/R and NPA motifs) to discriminate between silicic acid and arsenous acid. This possibility was largely unexplored until recent structural insights emerged from high-resolution cryo-EM and in silico mutagenesis studies (121). The potential to reengineer Si transporters to reduce As permeability while maintaining Si uptake marks a paradigm shift in plant nutrient and stress management strategies. Moreover, limited information exists on how transporter expression is modulated under simultaneous Si deficiency and As stress, or how root exudates and rhizospheric microbiota influence transporter functionality. These unexplored areas represent novel frontiers to enhance our understanding of Si-As dynamics.

Boorboori et al. (122) elucidated the mechanisms of Lsi1 regulating Si uptake, which influences As accumulation in rice seedlings. They discovered that the Lsi1 overexpression line (LE-OE) exhibited a superior capacity for Si absorption under hydroponic conditions compared to the wild type (LE-WT). Furthermore, the addition of Si to the LE-OE rice lines possessing the Lsi1 gene conferred enhanced As resistance relative to the LE-WT line. Khan and Gupta (123) demonstrated that compared to the control and Si treatments, the As(III)+Si treatment increased the expression levels of the OsLsi1, OsLsi2, and OsLsi6 genes involved in transporting As(III), but this increase was less pronounced than in treatments where As(III) was used alone.

### 3.4 As tolerance through improved antioxidant defense system and reduced uptake

A surplus of ROS, such as superoxide radicals (O2^+^·), hydrogen peroxide (H_2_O_2_), and hydroxyl radicals (·OH), is produced when toxic substances are present in plants, leading to oxidative stress (124). In the presence of ROS, various physiological processes are disrupted, including lipid peroxidation, protein oxidation, DNA damage, and the eventual stunting of plant development (125). Under As stress, Si supplementation dramatically boosts the activities of key antioxidant enzymes like superoxide dismutase (SOD), catalase (CAT), ascorbate peroxidase (APX), peroxidase (POD) and glutathione reductase (GR), as well as important non-enzymatic antioxidants like cysteine, ascorbic acid (AsA), and glutathione (GSH). Additionally, Si can induce heavy metal co-precipitation by surface adsorption by Si-rich tissues and thicken the cell wall, both of which impede heavy metal transport. Cui et al. (126) observed that treatment with SiO_2_ NPs could maintain the integrity of the cell, increase the thickness of the cell wall (77.4%) and the ratio of As in the pectin (19.6%). In addition, the pectin content, cation exchange capacity (CEC) and pectin methylesterase (PME) activity were also increased in the SiO_2_ NPs-pretreated cells, leading to a decreased degree of pectin methylesterification and an improved mechanical force of the cell walls. Silica-rich tissues (phytoliths) in rice can incorporate trace amounts of As, either through physical entrapment or surface adsorption (127).

Tripathi et al. (128) showed Si treatment enhanced SOD, GR and APX activities in rice plants exposed to As, resulting in lower ROS accumulation. Boorboori et al. (129) also found addition Si during As exposure significantly increased SOD, CAT, APX and POD activity and decreased MDA content in two different cultivars of rice. Geng et al. (130) observed that application of sodium silicate @ 168 mg L^−1^ increased SOD, CAT and POD activities along with elevated GSH and AsA contents implied the active involvement of ROS scavenging and played, at least in part, to Si-mediated alleviation of organoarsenic arsanilic acid (ASA) toxicity in rice. Li et al. (131) demonstrated As content in wheat shoots and grains decreased with the addition of Si-rich materials and maximum reduction of 16.2% and 17.8% in shoots and grains, respectively, was observed in rice husk biochar+2 g kg^−1^ bentonite treatment compared to control. Activity of GSH and AsA significantly increased with application of Si-rich materials with subsequent decrement in MDA content. However, As content in subcellular fractions of wheat shoots displayed no significant change after the Si-rich material addition. More similar studies have been summarized in Table 4.

**Table 4:** Impact of Si application on antioxidant defense mechanisms under As stress.

**Crop**	**Application rate of Si**	**Results**	**References**
Rice	1 mM silicic acid	Reduced H_2_O_2_, malondialdehyde (MDA) content and EC by 24.78–34.78%, 20.0% and 32.92–37.79%, respectively and increased SOD, CAT, APX and POD activity by 36.89–68.89%, 135.58%, 59.36–66.77% and 48.69–53.59%, respectively, in two different rice cultivars	(286)
Rice	Silicic acid @ 0, 0.5 and 1.0 mM	Decreased O_2_^−^·, H_2_O_2_, electrolyte leakage (EC) and MDA content by 11–16%, 9–10%, 13–17% and 13–18%, respectively	(128)
Maize	Si and biochar @ 100 mg kg^−1^ and 50 g kg^−1^, respectively	Combined application of Si and biochar significantly enhanced the antioxidant activities (SOD, POD, CAT, and APX) by 34.72, 23.12, 24.49, and 35.29%, respectively	(287)
Wheat	1.0 mM H_4_O_4_Si	CAT, POD, and GR activities significantly increased in roots under Si supplementation in As-stressed plants. In shoots, application of Si showed a significant increase in CAT activity compared with As stress.	(288)
Date palm	1 mM Na_2_SiO_3_	Enhanced accumulation of polyphenols (48%) and increased antioxidant activities (POD: 50%, PPO: 75%, GSH: 26.1%, CAT: 51%) resulted in a significant decrease in superoxide anion (O_2_^+^· : 58%) and lipid peroxidation (MDA: 1.7-fold)	(289)
*Brassica juncea*	SiO_2_ NPs @ 200 ppm	Significant reduction in oxidative stress markers, with H_2_O_2_ and MDA levels decreasing by 41% and 39%, respectively, and increased activities of antioxidant enzymes activity by 84% (SOD), 73% (POX), and 69% (CAT) along with 27% (proline content)	(290)

## 4 Agro-wastes

**Figure 7:**
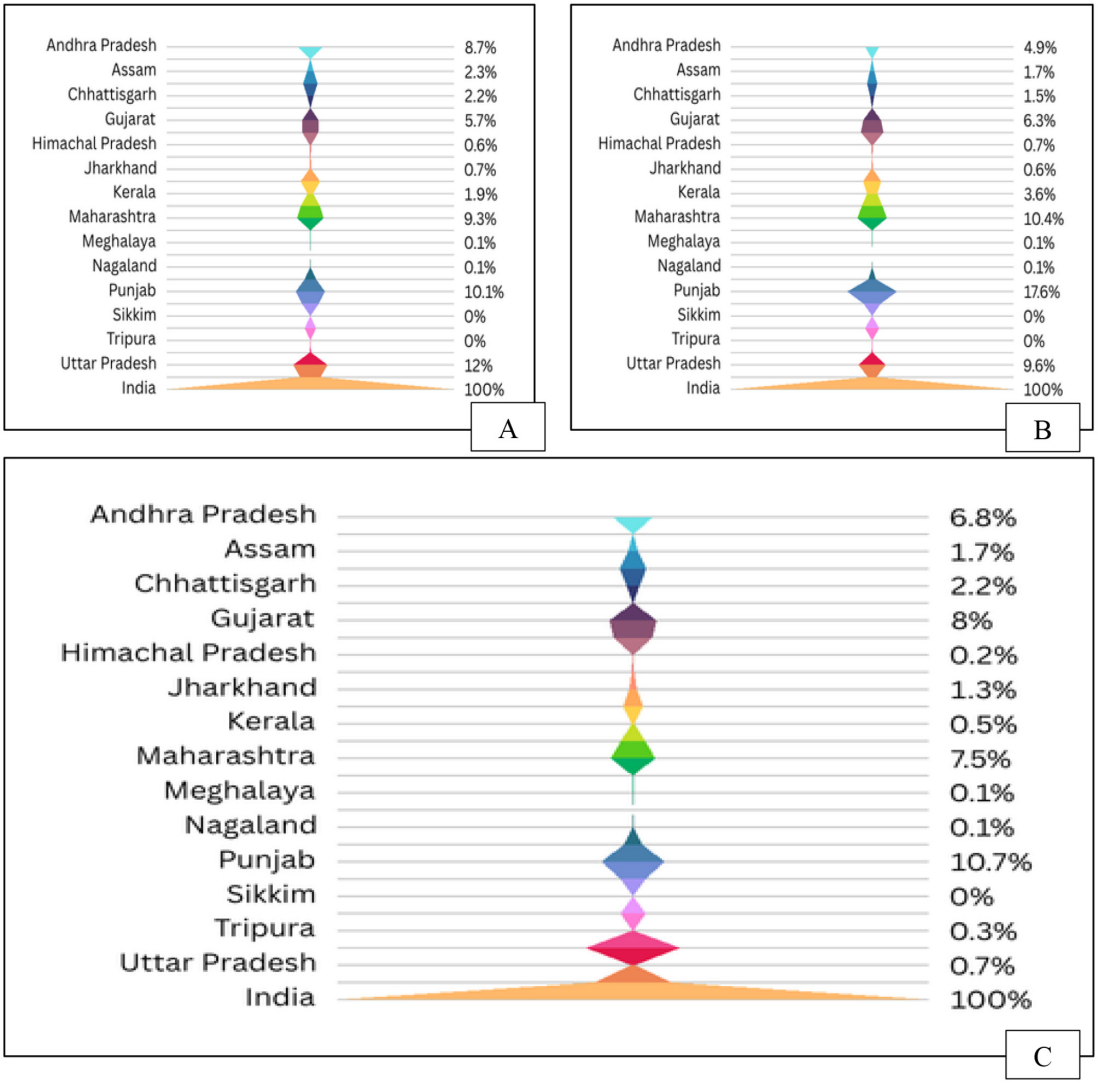
Crop residue dynamics in India: **(A)** State-wise residue generation, **(B)** Residue surplus availability, and **(C)** Residue burning status.

Agricultural wastes are residual byproducts from crop cultivation and initial processing of agricultural produce, including vegetables, fruits, dairy, meat, and poultry (132). These wastes encompass non-edible materials such as crop residues, forest litter, animal manure, and chemical remnants from fertilizers and pesticides (133). Generated through activities like seed production and livestock management, agro-waste poses serious environmental concerns, particularly when openly burned, contributing to air pollution and health risks (134). Post-harvest waste accounts for nearly 80% of total agricultural biomass, with burning still widely practiced. In India, Punjab, Maharashtra, and Gujarat are the leading states where extra residue is incinerated (Figure 7). Sustainable management requires conservation, recycling, and reuse strategies (135). Agro-wastes are categorized as field residues (e.g., stalks, stems), process residues (e.g., husk, bagasse, molasses), and commercial byproducts such as orange peel and oil cakes (136). Annually, millions of tons of agro-waste are generated worldwide, with over 90% in low-income nations being incinerated or discarded in open spaces, exacerbating environmental deterioration (137). Asian nations lead in the production of crop residues, particularly from silica-dense grains. India produces over 500 million tons of agricultural waste each year, contributing to a worldwide total of almost 1 billion tons (138). Due to escalating population pressures and food demand, nations such as India and China are encountering growing leftover surpluses. While usage and surplus fractions vary by crop type the surplus crop residue (Table 5) is improbable to meet potential demands; nevertheless, high-resolution spatio-temporal biomass availability may assist in overcoming current challenges in crop residue utilization (139).

**Table 5:** Global estimates of crop residue generation and surplus availability (MT yr^−^1) across selected countries [adopted from Sen et al. (291)].

**Country**	**Gross residue generation (MT yr^−1^)**	**Surplus (MT yr^−1^)**
India	500	140
	912	300
	682.6	178
	683	–
Bangladesh	72	–
	99.6	24.3
Indonesia	55	–
Myanmar	19	–
Afghanistan	9.7	2.2
Bhutan	0.4	0.1
Nepal	22.8	6.3
Pakistan	122.8	37.3
Sri Lanka	4.7	1.3
China	1,039.5	–
USA	488	–
World	2,445.2	–
	3,758	–

### 4.1 Si rich agro-wastes

Agricultural waste generation has increased steadily, driven largely by population growth, making it essential for environmental agencies to adopt strategies that minimize waste production. Recycling agro-wastes offers an effective means to reduce their adverse impacts on the environment and human health (140). Recent research focuses on using silica-rich waste materials to mitigate As bioaccumulation in plants (Table 6). Although Si is the most abundant element, its concentration in agro-wastes is lower than in primary minerals. Nonetheless, the vast quantities of agro-waste generated globally present a promising source for Si extraction (141). Alternative silica sources currently explored worldwide include rice husk, wheat husk, palm oil fuel ash, Miscanthus ash, e-waste, coal ash, reed ash, sedge ash, *Carex riparia*, sugarcane bagasse, bamboo leaves, natural clay, and ore tailings.

**Table 6:** Effectiveness of Si-rich agro-wastes and their derivatives in reducing As bio-accumulation in final produce.

**Raw material**	**Mechanism of arsenic mitigation**	**Effectiveness/efficiency (%)**	**Microbial mediation role**	**References**
Rice husk	Enhanced Si release to pore-water, Fe-plaque formation	25–50	Increased activity of soil microbes that express the As methyltransferase gene, arsM	(244)
Rice husk and husk ash	The competitive interactions between Si and As for plant uptake and sorption	36–58		(245)
Rice husk	Significantly more ferrihydrite and less goethite, thereby more As(III) associated with Fe-plaque	~40		(292)
Rice husk biochar and husk ash		20–24		(90)
Rice husk and husk ash	Increase in the mole ratio of porewater Si/As, indicating an elevated pool of dissolved Si to compete with As for root uptake by their shared transporters	~50		(249)
Fe-modified rice hull biochar	Decreased As/Fe ratio in root plaque	37–79	Reduced abundance of Fe(III) reducing bacteria by 24–64%	(250)
Rice straw biochar	Increased As solubilization in the porewater, functional groups of biochar capable of immobilizing As	41.4–57.5		(251)
Paddy straw compost with SSB	Reduced bioavailable As, higher Fe-plaque formation and presence of As uptake transporters in rice roots	34.2–53.2	SSB improved solubilization of Si from straw compost than its sole applicaiton	(10)
Charred rice husk	Increased the fraction of ferrihydrite in the root plaques	70.6	Increased the copy number of arsM in paddy soil, suggesting an increased capacity for arsenite methylation	(78)

#### 4.1.1 Rice husk

Rice is one of the most widely cultivated crops globally, with production surpassing 756 million tons in 2020. Milling generates approximately 20% of this yield as rice husk, a major by-product (142). Commonly discarded or used as fuel during parboiling, rice husk contains high levels of organic compounds such as lignin and cellulose, along with significant mineral content, particularly silica (143, 144). The high silica content has attracted interest for environmental applications, notably in reducing arsenic (As) toxicity in soils and plants. Rice husk ash (RHA), produced by combustion, typically contains 87–99% silica, depending on husk origin and burning conditions (143, 145). Quality and composition of RHA are influenced by soil type, climate, cultivation methods, and pre-treatment. RHA is characterized by high ash content compared to other biomass fuels, with properties such as high porosity, low bulk density, and large surface area, making it highly suitable for adsorption processes, including As mitigation. Silica in RHA occurs in both amorphous and crystalline polymorphs like, quartz, cristobalite, and tridymite, whose proportions depend on combustion temperature and treatment parameters (146). The crystalline structure formed is contingent upon the combustion temperature and treatment parameters. The extraction of amorphous silica generally entails acid leaching, succeeded by burning or pyrolysis to eliminate organic material and produce high-purity silica. This technique guarantees the synthesis of silica customized for catalytic, adsorption, and other sophisticated material applications (147).

The structural differences between amorphous and crystalline polymorphs influence adsorption affinity and binding mechanisms for As species [both As(III) and As(V)] in soil–water systems (148). Amorphous silica typically has a much higher specific surface area and more silanol (Si–OH) groups than crystalline quartz, enhancing As adsorption through ligand exchange or hydrogen bonding (149). The density and reactivity of Si–OH groups vary with polymorph type and surface treatment. More reactive surfaces (common in amorphous forms) facilitate stronger chemisorption of arsenate and arsenite ions. Crystalline silica is generally less reactive due to lower surface hydroxyl density, resulting in weaker As retention, unless weathering or surface functionalization creates active sites. Also, the point of zero charge (PZC) of different silica polymorphs influences As speciation and binding. For example, at pH above the PZC, surfaces become negatively charged, reducing electrostatic attraction for arsenate but allowing specific adsorption via inner-sphere complexes (150).

Utilizing RHA as a silica source enhances the value of an agricultural byproduct while fostering environmentally sustainable practices. The capacity to regulate silica polymorph formation by temperature and pre-treatment presents opportunities for specific applications in environmental research, such as arsenic remediation, water purification, and nanomaterial synthesis (151).

#### 4.1.2 Rice straw

Rice straw, a significant agricultural by-product, is produced in excess of 700 million tons each year after the rice harvest (152). Worldwide, around 20% of rice straw is employed, with more than 100 million tons incinerated each year (153), resulting in significant environmental and health issues, especially in nations such as India (154, 155). Rice straw possesses various potential applications, including animal feed, mushroom growing, energy generation, biochar, bioethanol, and biogas production; nevertheless, its elevated silica content constitutes a significant constraint. rice requires a significant amount of silica (10–12% of dry matter) (156) for mechanical strength and resistance to biotic and abiotic stressors (157). Silica exists in the dry matter of straw, predominantly as phytoliths, which enhance the plant's structural integrity (158). These silica-rich structures are integrated within the lignocellulosic matrix of the straw, consisting of cellulose (32–47%), hemicellulose (19–27%), and lignin (5–24%) (159, 160). Although it poses a hindrance to its application in certain sectors, the silica present in rice straw has significant environmental advantages. The integration of rice straw into soil using rice straw-based composites (RSBC) facilitates gradual Si release (159), hence augmenting nutrient availability, promoting plant development, and enhancing stress resilience, particularly in Si-deficient paddy fields (161). This promotes sustainable agriculture and aids in the attainment of Sustainable Development Goals (SDGs). Rice straw is a hybrid nanocomposite composed of cellulose and silica (SiO_2_), wherein silica nanoparticles serve as reinforcing agents within the plant's cellular matrix (162). Studies demonstrate that the majority of silica in straw is present in an amorphous state, predominantly located on the external surfaces of the sheath and stem (162). The incorporation of rice straw into circular bioeconomy methods, specifically for sustainable silica recovery and reuse, offers a practical approach to managing agricultural waste, mitigating environmental effect, and fostering resource-efficient farming systems (163).

#### 4.1.3 Sugarcane bagasse

Sugarcane (*Saccharum officinarum*) is crucial to the economics of numerous developing countries because of its importance in worldwide sugar production (164). Presently, Brazil is the foremost producer, accounting for approximately 36% of global output (165). Nonetheless, sugarcane processing produces substantial quantities of byproducts, including bagasse, straw, and cane tops (166), which present environmental disposal difficulties. Sugarcane bagasse is a viable feedstock for reducing arsenic (As) translocation in plants, owing to its availability, affordability, and substantial silica (Si) concentration. The buildup of silica in sugarcane is contingent upon the availability of silicon in the soil, which is absorbed by the roots in the form of silicic acid, thereafter transported, and deposited as amorphous silica throughout the plant tissues via transpiration. The silica concentration in sugarcane bagasse fluctuates according to species, soil conditions, fertilizer use, and growing methods. Sugarcane bagasse ash (SCBA) has both amorphous and crystalline silica, including quartz and cristobalite (167), with quartz occasionally included through sand adherence during harvesting (168). SCBA provides a sustainable alternative for silica production, facilitating waste valorization and circular economy frameworks. Enhancing recovery techniques, including response surface approach, guarantees high-purity silica (169) appropriate for diverse industrial applications while promoting ecologically sustainable resource management.

#### 4.1.4 Wheat husk

Wheat husk serves as a significant by-product in the wheat production process, with estimates indicating that around 1.5 tons of wheat husk are generated as solid waste for every ton of wheat produced (170). Conversely, wheat husk has frequently been incinerated or utilized as livestock feed and fertilizer. Consequently, the ash generated from burning wheat husk (WHA) can lead to significant environmental issues due to the emission of substantial amounts of harmful pollutants. To mitigate this significant environmental issue, studies have been undertaken regarding the utilization of WHA as a renewable, cost-effective, and environmentally friendly source of amorphous silica, considering the high silica content found in WHA (171). The wheat husk primarily consists of cellulose (23–42% by weight), hemicellulose (18–21% by weight), lignin (14–28% by weight), and starch (9–19% by weight); lignin renders it a possible source of silica/lignin hybrid minerals (172). Various researchers conducted analyses on the elemental silica content, determining it to be approximately 2.1% (weight basis) to 2.57% (weight basis). Sodium silicate is a compound that serves as a precursor to Si. Its extraction from ashes presents an alternative method, as traditional production processes demand significant energy, typically sourced from quartz sand combined with sodium carbonate at 1,300 °C (173). Biosilica-based materials derived from wheat waste may serve as secondary products that enhance the value of agricultural crops. Furthermore, silica with varying properties, such as nano silica and meso/macro porous silica, can be efficiently produced from wheat husk tailored to its specific application (174). The ash content of the wheat husk and spike exceeds 20%, comprising 86% SiO_2_, which is ascribed to the type of fertilization applied (172). Terzioglu et al. (175) determined that an ashing temperature of 1,000 °C yields the highest SiO_2_ content; however, this temperature cannot be regarded as the optimal ashing temperature due to the irrecoverable structure of silica (cristobalite). Wheat husk phytoliths are spherical (14–22 μm diameter) and oblong (18–40 μm length, 12–18 μm width) in epidermal cells and consist of a silica shell and the plant cell's organism core (176). Wheat husk possesses a higher concentration of surface Si, rendering it a more viable Si source for the remediation of As toxicity (138).

#### 4.1.5 Bamboo leaf

Bamboo is one of the most important non-wood forest products worldwide, valued for its rapid growth and diversity, particularly in subtropical regions of Asia, Africa, and Latin America (145). It is widely used in construction, household items, pulp, paper, textiles, and handicrafts. However, only about 40% of harvested bamboo is effectively utilized, with 50–80% discarded as agro-industrial waste (177). While bamboo stalks are the primary raw material, leaves are generally treated as waste. These leaves can be used as a fuel source, producing considerable quantities of bamboo leaf ash (145). Although agro-wastes like rice husk, corn cob, and sugarcane bagasse are well-known silica sources, bamboo leaves remain underutilized, despite being an abundant, low-cost, and commercially untapped source of high silica content. The ash from bamboo leaves contains a significant silica content, ranging from approximately 75.90% to 82.86%, as indicated by Olawale (178). Setiadji et al. (179) successfully extracted 81.76% pure amorphous silica from bamboo leaf ash using an alkaline solvent.

#### 4.1.6 Corn cob

Corn cobs, an agricultural byproduct of maize—a major grain crop cultivated globally—are composed primarily of cellulose and lignin, with notable mineral content including silicon (Si, 0.133 wt%) (180, 181). Upon combustion, corn cob ash (CCA) contains over 60% silica by mass along with trace metallic elements (182). Produced as a fine powder, CCA requires no further grinding, making it a highly cost-effective raw material for silicate, silica, and silica nanoparticle production (183). While corn cobs have been extensively studied for uses such as enzyme production, protein extraction, adsorbents, fuels, and cement manufacturing, limited research has explored CCA for silica extraction and applications. Chanadee and Chaiyarat (184) demonstrated that sweet corn cobs (Zea mays *saccharata* L.) yield optimal silica powder at a combustion temperature of 600 °C. XRD analysis confirmed its amorphous structure, FTIR identified silanol and siloxane functional groups, and XRF revealed a silica content of 46.9% (185). These findings highlight CCA as a promising, low-cost, and underutilized silica source for industrial and environmental applications.

#### 4.1.7 Reed ash

*Phragmites australis (Cav) Trin. ex Steud*, commonly known as common reed, is a native perennial plant found in wetlands globally, primarily utilized as a domestic fodder (186). It can be utilized for several applications, including paper manufacture, construction materials, feed, phytoremediation, electricity generation, energy supply, and bioethanol. Aquatic common reed significantly contributes to aquatic habitats by serving as a natural cleanser through its phytoremediating properties and mitigating river erosion (82). Currently, the common reed is recognized as a significant environmental issue, as its adaptability to various environments obstructs the growth of other ecologically vital plant species. Notwithstanding the various applications of reed, it has been utilized in certain regions globally as a financially sustainable biomass for energy generation, as noted by Kobbing et al. (187). Subsequently, the incineration of common reed for energy generation results in the formation of common reed ash (CRA) as the primary by-product (188). CRA possesses a significant SiO_2_ content and offers a distinctive opportunity to serve as a cost-effective and plentiful source of amorphous silica (145) for the environmentally conscious mitigation of As toxicity.

## 5 Microbial mediated solubilization of Si

Although constituting 27% of the Earth's crust and ranking as the second most abundant element, the limited solubility of most Si forms inhibits their absorption by plants (189). Si exhibits a notable affinity for oxygen; consequently, it predominantly occurs in nature as silicates (SiO_3_), a form that is not readily absorbable by plants (17). Aluminosilicates, ferromagnesian silicates, silicon dioxides, amorphous silica, clay, feldspar, and mica are all examples of compounds that fall under the umbrella term “silicates.” Other silicates contain iron, calcium, sodium, potassium, or sodium, and ferromagnesian silicates include amphiboles, olivine, and pyroxenes. Silicas make up more than 90% of the Earth's crust and are present in substantial amounts in sedimentary, igneous, and metamorphic rocks as well. Depending on the soil's pH levels, Si can also appear as silicic acid (190). The release of Si into the soil by weathering or dissolution is necessary for plant uptake (191). Along with water, plants absorb orthosilicic acid, a soluble form of Si. According to Klotzbücher et al. (192), monosilicic acid is produced when soil nutrients are depleted, Si-containing minerals weather, and irrigation is used. Si fertilizers, in contrast to more conventional fertilizers, are expensive and scarce, making them out of reach for most farmers. Hence, Si fertilizers are rarely used, especially in developing countries (17). Reusing materials with Si concentrations from mining, agriculture, and construction and demolition can lead to the production of silicate fertilizers with long-term economic viability (193). Thakral et al. (194) reported that the concentration of Si in the soil solution is significantly affected by the solubilities of both primary and secondary minerals. Soil applications involving biochemical and physicochemical treatments can speed up the solubilization of these chemicals, with microbial activity being the most important factor in biochemical action (17).

Microorganisms are recognized for their ability to breakdown and mobilize minerals in the soil (195). Numerous investigations have established that microorganisms isolated from silicate mineral surfaces weather various silicates (196, 197). This signifies the crucial function of silicate-solubilizing microorganisms (SSM) as biofertilizers in the solubilization of silicates and phosphates (198, 199). Microorganisms are prevalent in soils, although only a limited subset is capable of solubilizing insoluble silicates. Plants and microflora are known to generate chelating ligands, modify soil physical properties, and influence the dissolution and mobilization of soil silicate minerals (199). Among microorganisms, plant-associated bacteria, fungi, actinomycetes have been documented to facilitate the dissolution of silicates and expedite the release of Si into the plant-soil system through bio-weathering processes.

### 5.1 Silicate solubilizing bacteria (SSB)

Microorganisms such as *Burkholderia, Bacillus, Pseudomonas*, and *Enterobacter* have been documented to solubilize various types of silicates, including magnesium silicate, quartz, feldspar, and other insoluble silicates (Table 7). SSB is primarily located in soil, water, sediment, mineral ore, weathered rocks, and the rhizosphere of plants, where it plays a crucial role in regulating the biogeochemical cycle of Si (200). Vasanthi et al. (201) indicated that a considerable amount of SSB linked with phyto-sil, muscovite, and calcium aluminosilicate suggests that these minerals preserve them from their natural sources of extraction. The clarification provided indicates that the ratio of SSB associated with a mineral does not align with its silica concentration. For instance, muscovite, which contains 21% silica, displayed a higher proportion of SSB compared to phyto-sil, which has 78% silica. This observation contrasts with quartz at 98%, talc at 54%, and feldspar at 45% silica. The findings clearly demonstrate a notable difference in the overall bacterial presence within soil or silicate minerals compared to the SSB.

**Table 7:** Silicate solubilizing bacteria isolated from different cultivars and their role.

**Sl. no**.	**Agro-ecology**	**Silicate solubilizing bacteria**	**Plant cultivar**	**Source of isolation**	**Medium of isolation**	**Source of silicate used for isolation/characterization**	**Focused area of interest**	**References**
	Daegu, a city of Gyeongbuk Province, Republic of Korea	*Burkholderia eburnea*	*Oryza sativa* L. cv. Dongjin	Rice rhizosphere	Silicate medium	Magnesium trisilicate	Silicate solubilization, IAA production, ↑ plant growth, ↑ Sie uptake and deposition	(18)
	Institute of Natural Sciences and Mathematics, Ural Federal University, Ekaterinburg, Russia	*Bacillus* Species	*Brassica juncea (L.)*	Clay substarte	Zak-Alexandrov medium	Sodium silicate	Structural and functional parameter of photosynthetic apparatus	(293)
	Microbiology and Environment Laboratory, the Indonesian Research Institute for Biotechnology and Bioindustry, Bogor	*Burkholderia cenocepacia* KTG, *Aeromonas punctata RJM3020* and *Burkholderia vietnamiensi* ZEO3	_	Sandy soil	Bunk and Rovira medium	Magnesium trisilicate and quartz	Production of citric, acetic and oxalic acid; ↑ solubilization of silica	(294)
	Teaching Farm of Fujian Agriculture and Forestry University, Fuzhou, China	*Aeromonas, Bacillus, Cellvibrio, Ensifer, Flavobacterium, Microbacterium, Paracoccus, Pseudomonas, Rhizobium*, and *Streptomyces*	*Zea mays* L. cv. Yuebai	Earthworm gut and surrounding soil	Orthoclase feldspar	Aleksandrov's medium	Silicate weathering and availability to plants	(295)
	Division of Microbial Technology, CSIR-National Botanical Research Institute, Lucknow, India	*Pseudomonas* and *Bacillus (Sphingobacterium* sp*., B. amyloliquefaciens)*	*Oryza sativa* cv. Jayanti	Rhizospere	Magnesium trisilicate, talc, and feldspar	Si solubilizing media (NBRISSM) containing feldspar as silicate	↑ Si uptake, ↓ disease severity, and antioxidative enzyme activities	(242)
	Gyeongbuk, South Korea	*Enterobacter ludwigii*	Rice mutant *Waito-C* and rice cultivar ‘*Hwayoungbyeo*	Paddy soil and forest soil samples	Magnesium trisilicate	Glucose agar medium	Potential Si and phosphate bio-fertilizer	(296)
		*Bacillus mucilaginosus*		Rhizosphere soil	Magnesium trisilicate	Bunt and Rovira medium		(297)
	Puducherry, India	*Bacillus flexus, B. mucilaginosus, B. megaterium and Pseudomonas fluorescens*	_	Soil samples from red soil, plantation soil, sea sand, pond sediment, sea water	Magnesium trisilicate, feldspar, calcium aluminosilicate, sodium aluminosilicate, talc, muscovite, illite and quartz	Bunt and Rovira medium	The dissolution of silica in solution functions as a nutrition for living organisms.	(201)
	Longshan (Nanjing, China)	*Rhizobium tropici*	_	Weathered rocks	Feldspar and biotite	Solid K-limited medium (KLM)	↑ Si and K concentrations	(298)
	Besut, Terengganu, Malaysia	*Serratia marcescens and Pseudomonas aeruginosa*	*Oryza sativa* var MR219	Rhizosphere soil	Magnesium trisilicate	Magnesium trisilicate media	↓ chemical application in rice sheath blight	(49)
	Fujian Agriculture and Forestry University, Fuzhou, China	*Kosakonia sp*.	*Zea mays L*.	Bryophyte Hypnum plumaeforme rhizoids	Feldspar and silica	Aleksandrov medium	Si availability in the soil, Si uptake and plant growth	(203)
	ICAR- IRRI farm, Rajendranagar, Hyderabad, Telangana, India	*Rhizobium sp*.	*Oryza sativa* genotype BPT 5204	Rhizopshere soil	Bentonite, calcium aluminio silicate, fuller's earth, kaolin, magnesium trisilicate, potassium alumino silicate and quartz	Bunt and Rovira medium	↑ Rhizosphere available Si concentration.	(224)
	Chinese Academy of Agricultural Sciences	*Bacillus mucilaginosus*	_	Chinese Academy of Agricultural Sciences	Mica and feldspar	Silicate medium	Decomposition of silicate minerals by the becterium	(299)

The rhizosphere of crop plants such as rice has been extensively studied for the isolation of SSB due to the significant Si need and uptake by rice plants. Comparable initiatives have been implemented with numerous other Si-rich accumulator plant species (200). Tropical forests, especially bamboo forests, are recognized as significant sources of soluble Si in rivers. This is mostly attributable to the significant accumulation of Si in bamboo leaves, perhaps due to the activities of soil-Si bacteria. Nevertheless, minimal attempts have been made to discover SSB inside bamboo rhizosphere or forest environments. Recent reports indicate Si buildup in 456 distinct plant species cultivated under same soil conditions (202). Hu et al. (203) identified a *Kosakonia* genus SSB from the rhizomes of *Hypnum plumaeforme*, which promotes Si absorption and accumulation in maize, hence increasing growth. The investigation of SSBs offers a cost-efficient and eco-friendly approach to augmenting plant nutrition in Si, phosphorus, and potassium, consequently raising agricultural yields (201, 204).

### 5.2 Silicate solubilizing fungi (SSF)

The majority of the literature is based on the population and variety of SSB, whereas SSF has been minimally investigated. The fungal species *Aspergillus niger, Trichoderma* sp., *Beauveria caledonica*, and *Serpula himantioides* have been examined for their ability to solubilize silicates (205). Two SSF isolated from soil were screened and identified as *Penicillium limosum* and *Bipolaris sorokiniana* (206).

### 5.3 Mechanism involved in silicate solubilizing activity observed in microbes

The extraction of microbiological nutrients from insoluble silicates depends on a conventional geochemical process known as bio-weathering (200). In this process, living things break down soil minerals and bring them to the surface. A wide variety of saprophytic bacteria, actinomycetes, and fungi are the principal agents of bioweathering. The growth of plants is supported by these bacteria because they dissolve important nutrients for plant-soil interactions. Plants are able to absorb and use newly formed nutrients because bio-weathering is the main process that transforms polymerized silica into monomeric forms (207). The bond strength between Si and its neighboring components determines how easily the bonded Si can be released from the framework (208). An example of a material that shows resistance to dissolving even when subjected to high temperatures and pressures is SiO_2_ polymers (82). Materials such as quartz, silica, and phytoliths can only be dissolved through proton action and mineral-bound cation exchange, while metal-bound silicates require a coordinated shift in pH and ligand attack (200, 209). Some bacterial species may have varying solubilization capacities depending on the mineral supply. Biogenic materials like siliceous earth, diatomaceous earth, rice straw, and rice husk, insoluble inorganic silicates of potassium, magnesium, and aluminum, and silicate minerals like biotite and feldspar are all potential sources of soluble silica that these bacteria can release (17).

**Figure 8:**
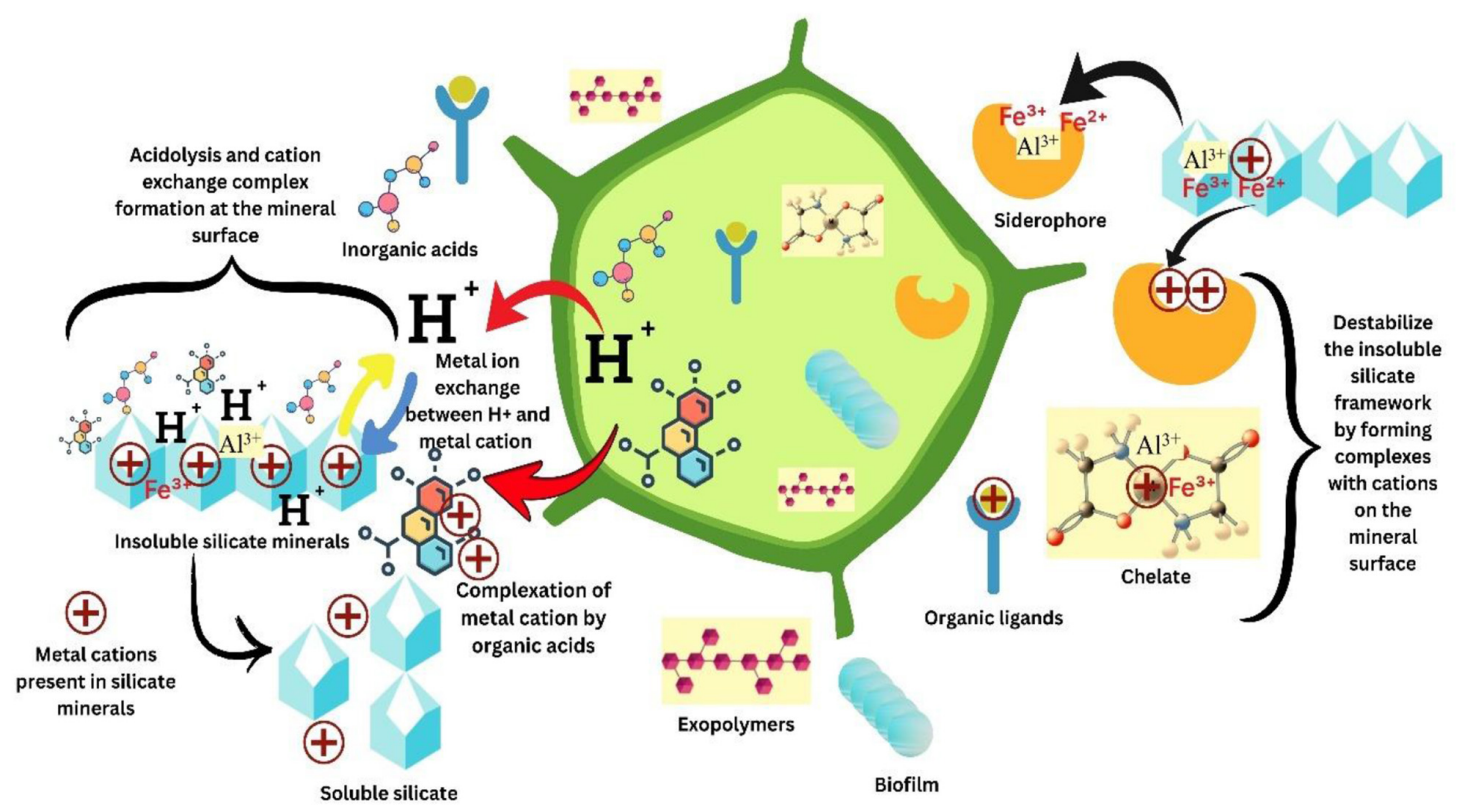
The molecular mechanisms behind the silicate solubilising activity reported in bacteria. The silicate solubilisation process is facilitated by several processes, including the reduction of pH through the production of organic and inorganic acids, the displacement of charged particles at mineral surfaces, and the synthesis of microbial metabolites, enzymes, and exopolymers.

In the most fundamental concept of silicate solubilization, bacteria employ a number of mechanisms to facilitate a multi-step process. According to many studies (200, 210–212), the process begins by replacing protons on the mineral surface with charged cations such as K^+^, Na^+^, and Ca^2+^. Then, hydrolysis occurs and the silica species is detached from the framework. In order for microbes to break down and dissolve silicates, they are thought to employ a number of interconnected processes (Figure 8), such as (i) lowering the pH through the production of inorganic and organic acids, (ii) synthesis of chelating metabolites, and (iii) engaging in nucleophilic attack and exchange reactions (213). The primary mechanism observed is the acidolysis occurring in the vicinity of microorganisms (214).

#### 5.3.1 Changes in pH due to organic acid production

An important geochemical phenomenon, the interaction between organic acids and mineral surfaces has been studied extensively for decades, especially LMOAs (215). LMOAs are frequently present in rhizosphere soils, particularly in the layer immediately adjacent to the soil-root contact (216). The breakdown of plant roots, fungal remnants, and other organic components mostly produces these acids (217). Lazo et al. (218) found that organic acids and their anions can accelerate mineral weathering. Kong et al. (216) suggested that organic acids can form complexes with soil elements due to the presence of carbonyl and hydroxyl functional groups. According to Drever and Stillings (219) and Lazo et al. (218), there are three primary processes that impact mineral weathering caused by organic acids: (a) changes in solution ion speciation, (b) adjustments in solution saturation relative to the mineral, and (c) disturbance of the dissolution reaction from equilibrium. Casey et al. (220) described mineral bio weathering as an acid-base process involving bridging oxygens that occurs when hydroxyl or hydrogen ions are adsorbed onto the surface of minerals. Consequently, the amount of hydroxyl ions and protons on the surface plays a pivotal role in the release of Si. In the microenvironment, that microbes create surrounding a mineral, protons and other organic and inorganic compounds are secreted, which aid in the breakdown of silicates (200). As a consequence of released H^+^ exchanging cations within the silicate framework, a cation exchange complex might form on the surface of the material. Acidolysis is further expedited when bacteria release both organic and inorganic acids (221). A high proton content and an acidic environment make cation replacement easier (222, 223). A change in the dissolution rate, away from equilibrium, causes silicates to dissolve more quickly in an acidic environment (200). The diversity of microbes determines the specific organic acid that is emitted. There are a number of organic acids that have been found to dissolve silicates in media that contain quartz, feldspar, and magnesium trisilicates as sources of Si. These acids include maleic, succinic, fumaric, gluconic, tartaric, and hydroxy propionic acids (201, 224). In addition to the organic acids generated by bacteria, the breakdown of organic matter generates NH_3_, H_2_S, and CO_2_. These byproducts are easily bio-converted into inorganic acids by microorganisms such as *Thiobacillus, Nitrosomonas*, and *Nitrobacter* (200). It has been suggested that the creation of ammonia and amines might cause an increase in the pH of the surrounding environment, which in turn affects silicates. This suggests that the production of alkali could be a way for silicates to be solubilized (225). According to Kutuzova (225) and Rajabipour et al. (226), silicates can be influenced by changes in environmental pH caused by the generation of ammonia and amines, suggesting that alkali production could be a way for silicates to be solubilized. In a study conducted by Sheng et al. (227), it was found that Bacillus globisporus Q12, a type of bacteria that can dissolve silicates, was able to dissolve K and Si in silicate minerals like muscovite, biotite, and feldspar. The researchers found that organic acids, specifically acetic and gluconic acids, were the most effective in this process. In a different study, Sheng and He (228) found that SSB-driven illite and feldspar help produce organic acids like malic, tartaric, gluconic, citric, oxalic, succinic, and 2-ketogluconic acids. When it comes to solubilizing potassium or silicates, tartaric acid is by far the most common agent. The local environment and ionic composition can be altered by microbial deposition near silicate sources; however, our understanding of the consequences of ionic strength is lacking on-exchange reactions taking place on mineral surfaces might be hindered by elevated ionic strength (229). Speciation on the surface is affected by changes in ionic strength because of the double layer effect. When the ionic strength increases, the surface charge becomes more positively charged at pH levels below pH_zpc_ and more negatively charged at pH levels above pHzpc. As a result, it speeds up the breakdown process (230).

#### 5.3.2 Synthesis of chelating metabolites

In addition to acidifying and improving the solubility of silicates, Organic and inorganic acids can protonate and hydrolyze them, while concurrently complexing with the cationic components of silicates, making them possible chelating agents (201). Microbial metabolites such as extracellular enzymes, siderophores and other reaction byproducts play a significant role in silicate dissolution. These microbially excreted metabolites possess metal complexing properties that can bind with aluminum and iron in silicates, eventually destabilizing the silicate framework, thereby increasing the solubility of silicates. Drever and Stillings (219) reported the formation of oxalate complex due to the reaction of oxalic acids with Fe and Al. This in turn reduces the chemical activity of the cations in the silicate framework. The dissolution of silicates resulting from the production of keto-gluconic acid by bacteria, which complexes and chelates with metals, has also been reported (201, 231).

Siderophores are low molecular weight organic chelators characterized by a high and specific affinity for Fe (III). Siderophore biosynthesis is regulated by iron concentrations, and siderophores facilitate iron uptake in microbial cells (232). Bacteria, such as cyanobacteria, fungi, and plants that utilize phytosiderophores, synthesize siderophores in environments with low Fe3^+^ concentrations (17). Siderophores produced by SSB can solubilize Si by extracting iron from silicate minerals, as evidenced in hornblende degradation (233, 234). Phosphate-solubilizing bacteria (PSB) can also solubilize silicates via siderophores, potentially affecting the solubilization of Si and phosphorus from rocks (235).

#### 5.3.3 Nucleophilic attack and exchange reactions

Stumm (230) found that when ligands are included in the coordination sphere of metal ions, the reactivity of the other molecules of coordinated H_2_O is enhanced. In general, the coordinated ligand's σ electron-donating, nucleophilic capacity causes the water exchange rate to increase. The water exchange rate is increased by several orders of magnitude when an OH^−^ ion attacks a hexa-coordinated aquo metal ion nucleophilically (230). This means that the surface functional groups have been deprotonated, which increases the reactivity of the –Si–O-bond. The dissolution process at the surface of the mineral, which is aided by OH-bonding, deprotonation, or ligand complexation, is known as depolymerization, or the dissociation of a Si–O–Si link. Surface hydroxyl group replacement ligands can bind nucleophilically with metal ions in the surface lattice to form surface crystalline bonds. Dicarboxylic acids, hydroxy carboxylates, diphenols, EDTA, and NTA are ligands that contain functional groups with two or more donor atoms; these ligands can form bi- or multi- dentate mononuclear surface chelates, which are very efficient. The surface lattice is negatively charged and surface protonation is enhanced by the presence of certain ligands. As the surface concentration of ligand increases, so does the rate of ligand-assisted dissolution. The surface metal centers can be released into solution more easily when a bi- or multi- dentate ligand coordinates inside a mononuclear inner-sphere surface complex, which aids in ligand-facilitated dissolution (236). Factors of paramount importance include the surface chelate size and the quantity of donor atoms coordinating to a particular surface metal center. When it comes to improving the rate of dissolution of Al-minerals, Furrer and Stumm (237) reported that the five-membered surface chelate ring of oxalate is better than the six-membered rings of salicylate and malonate, as well as the seven-membered rings of succinate and phthalate. Monodentate organic surface complexes have a negligible effect on the dissolution of σ-Al_2_O_3_. Complex formers generally form rather weak surface complexes on silica surfaces; nevertheless, the nucleophilic citrate and oxalate enhance the dissolution rate of quartz (238).

#### 5.3.4 Exopolymers and enzymes

Silicate-solubilizing bacteria generate extracellular proteins and polysaccharides that create biofilms surrounding their colonies (239). These biofilms facilitate microbial adhesion to mineral surfaces and affect mineral dissolution. They establish a micro-environment that minimizes the loss of protons, ligands, and organic acids (200). Biofilms possess water retention properties, hence promoting mineral weathering. Elements of the bacterial cell membrane, including lipopolysaccharides, peptidoglycan, and teichoic acids, can interact with silicate ions for solubilization (240). Engineered gluconic acid synthesis and excellent dissolving of poorly soluble calcium phosphates were achieved by cloning the gabY gene from *Pseudomonas cepacia*. This process shed light on the genetic principles of mineral solubilization (241). When it comes to solubilizing Si, Bist et al. (242) have shown that acidic phosphatase activity and organic acid generation are functionally related. New developments in high-throughput whole genome sequencing have made it possible to identify the genes that play a role in the metabolic pathways of acids, exopolymers, membrane transporters, and silicate-solubilizing ligands (200). For weathering of silicate minerals, these phenomena can be studied in greater detail.

## 6 Methods of Si-rich agro-wastes applications

Si-rich agro-waste, derived from crop residues such as rice husk, rice straw, wheat straw, sugarcane bagasse, corn stover etc. provides a sustainable source of plant-available Si to enhance crop productivity and stress tolerance (243). The effectiveness of these residues depends not only on their Si content but also on the application method, which influences Si solubilization, nutrient dynamics, and interaction with soil contaminants such as As.

### 6.1 Direct soil incorporation of raw biomass

Unprocessed crop residues are chopped and incorporated into soil before planting. Their subsequent decomposition releases soluble Si (H_4_SiO_4_) via microbial mineralization (191). Si accumulates in the rhizosphere, enhancing uptake by plant roots. This process although enhances soil organic carbon and microbial activity, the release of Si is slow and initial nitrogen immobilization during decomposition may occur, therefore, suitable for long-duration crops. Seyfferth et al. (244) observed that rice husk incorporation to soil (1% w/w) decreased grain As by 25–50% and straw As by at least 50%, and increased straw and husk Si by 25–60% without affecting yield in three different rice cultivars. Mamud et al. (245) conducted a study in Meghna Estuarine floodplain of Bangladesh which is known for its As laden groundwater and found out that in the Pleistocene terrace soils, fresh rice husk (1% w/w) reduced As in grain, husk, and straw by 36–40%, 36–41%, and 42–45%, respectively and in the Holocene floodplain soils, by 39–45%, 55–58%, and 50–51%, respectively.

### 6.2 Application of agrowaste in combusted form

Depending on the process of combustion two types of amendment can be derived from agrowastes. Open-air burning or controlled combustion of biomass in presence of oxygen at higher temperature produces ash such as RHA or sugarcane bagasse ash, can be applied directly to the field (246). Ash contains amorphous silica which dissolves in water to release monosilicic acid (173). Ash can also co-precipitate As and bind other heavy metals. Biomass, when pyrolyzed at controlled temperature (350–600 °C) and oxygen environment, produces biochar. Biochar retains Si in a reactive form and serves as a slow-release Si source (247). It also improves cation exchange capacity and As adsorption due to its porous structure. Several studies show the advantage of ash and biochar over direct incorporation of residue. Penido et al. (248) observed that both RHA and rice straw ash (RSA) amended soils had low As levels at or less than 0.2 μM L^−1^. Soils supplemented with fresh husk (FH), whether whole or powdered, exhibited marginally increased solution-phase Concentrations varied from 0.2 to less than 0.6 μM L^−1^ for FH whole and from 0.2 to 0.5 μM L^−1^ for FH powder amended soils. The solution phase exhibited concentrations approximately nine times greater in fresh straw amended soils compared to those amended with FH, RSA, or RHA, ranging from 1.0 to 1.8 μM L^−1^. Leksungnoen et al. (90) evaluated biochar and ash formed from Si-rich rice husk and showed that rice husk biochar and RHA (64% w/w) effectively reduced inorganic As buildup in rice grain to 0.27–0.29 mg kg^−1^, representing a 20–24% reduction compared to the control. Moreover, RHA substantially reduced grain-As(V) concentrations. Wang et al. (249) documented a 15.9–40.5% reduction in pore water As from tillering to harvest of rice, attributed to the application of Si-rich RHA in comparison to rice husk. The sequestration of As in the soil solid phase and root plaque rose by 8.0% and 26.9% with the application of RHA, likely due to the co-precipitation of iron and As facilitated by the liming effect of RHA, which was associated with a significant reduction in As transit. The inorganic As content in white rice diminished from 0.36 mg kg^−1^ in the control group to 0.24 mg kg^−1^ with rice husk and 0.17 mg kg^−1^ with Si-rich RHA, underscoring the efficacy of Si-rich RHA compared to rice husk. Kumarathilaka et al. (250) found that iron-modified Si-rich rice hull biochar (Fe-RBC) under intermittent flooding reduced As buildup in rice roots, shoots, husks, and unpolished grains by 62%, 37%, 79%, and 59%, respectively, in comparison to the standard flooded treatment. Limmer et al. (78) found a reduced concentration of straw and root As from 0.65 and 11.2 mg kg^−1^ in husk treated plants to 0.57 and 7.4 mg kg^−1^, respectively in charred rice husk treated plants. Maximum reduction (70.6%) in dimethylarsinic acid content in panicles was found in high-Si rice straw biochar applied pots followed by low-Si rice straw biochar applied pots (60.2% reduction) as compared to control (251).

### 6.3 Composted or co-composted Si-rich biomass

Crop residues are composted alone or co-composted with nitrogen-rich material (e.g., animal manure) to produce stable organic fertilizer enriched with Si. Microbial culture like PSB, SSB, and potash mobilizing bacteria can also be added for faster release of nutrients. Composting enhances Si bioavailability by degrading the phytolith matrix and increasing microbial solubilization (252). Khanam et al. (10) observed rice straw compost (RSC) significantly reduced (32.5% reduction) bioavailable As (NaHCO_3_ extractable) content compared to other amendments. The combination of SSB+RSC caused a further reduction by 38.7% in soil. The application of SSB+RSC resulted in a greater reduction in roots, shoot, and grains with the value of 49.4%, 34.2%, and 53.2%, respectively. The SSB+RSC treatment resulted in the highest transfer rates of As from soil to root and from shoot to grain were found to be the lowest (3.4 and 0.16, respectively) with SSB+RSC followed by RSC (4.0, 0.20). Yamaguchi et al. (253) reported that in lime + 2,250 g m^−2^ rice compost applied site, the pseudototal As concentration in the soils after 97 cycles of rice cultivation was approximately 60% of that in the plots without annual compost application. As concentration in the shoots and panicles of rice plants was consistently lowest in the lime + 2,250 g m^−2^ rice compost applied plots for last 6 years (92nd to 97th cropping).

### 6.4 Extraction of silica from agrowaste

Silica (SiO_2_) is well known as a precursor for many applied forms of Si like calcium silicate, sodium silicate, silicic acid, silica nanoparticles (SiO_2_ NPs), silica gel etc. These materials can be directly incorporated by soil applied or foliar spray and get rapidly absorbed through roots or stomata. Production of SiO_2_ from agricultural wastes can be accomplished in three different ways: chemical treatment, thermal treatment, or microbiological treatment (254). Different extraction methods with their final product has been depicted in Table 8.

**Table 8:** Different methods of extracting silica from agro-waste [adopted from Seghir et al. (254); Setiawan and Chiang (243)].

**Raw material**	**Extraction method**	**Extraction condition**	**Product**	**Average silica particle size**	**Silica purity (wt %)**	**References**
Rice husk	Hydrothermal extraction	Ethanol 180 °C, 0.1 MPa, 24 h	Amorphous silica	101 m^2^ g^−1^ of specific surface area		(300)
Sorghum husk	Hydrothermal extraction	1 M HCl, 120 °C, 0.1 MPa, 2 h	Amorphous silica	spherical, saddle, and dumbbell shape		(301)
Rice husk	Combustion in muffle furnace	600 °C, 2 h	Amorphous silica	0.50–0.70 μm	95.77	(144)
Rice straw	Combustion	500 °C, 8 h	Amorphous silica		72.60	(302)
Coconut husk	5N H_2_SO_4_ treatment, Combustion	700 °C, 3 h	Crystalline silica		91.76	(303)
Pine cone	3M H_2_SO_4_, Thermal decomposition	600 °C	SiO_2_ NPs	37 nm		(304)
Rice husk ash	Acid precipitation method	HCl washing before extraction, 60 °C	Amorphous silica	0.50–0.70 μm	99.2%	(144)
Rice husk ash	Acid precipitation technique	80 °C	Amorphous silica	10–15 nm	98.9%	(305)
Paddy straw	Acid precipitation	Acid wash, 37 °C	Nano-silica	15–20 nm		(306)
Wheat straw	Leaching in a 10% (v/v) HNO_3_ and calcination	4:1 (v:v) mixture of nitric and sulphuric acid washing, 400–700 °C	Amorphous hydrated silica	75–320 nm		(307)
Sugarcane bagasse (SCB), corn stalk (CS), and rice husk (RH)	Calcination in a Thermolyne muffle furnace	1M HCl washing, SCB was calcined at 950 °C, 4 h, CS at 550 °C, 4.5 h, and RH at 500 °C, 4 h	Crystalline SiO_2_ for SCB and CS and amorphous for RH	25.0, 6.84 and 3.79nm for SCB, CS and RH, respectively	30.21, 29.51 and 31.4% for SCB, CS and RH, respectively	(308, 314)
Olive stone	Alkali leaching process	10% HCl wash, Ambient temp	Crystalline silica	15–68 nm		(309)
Olive stones	Alkali leaching extraction method	Acid wash, 900 °C	Crystalline silica	15–68 nm		(310)
Cassava periderm	Sol–gel method	0.1 M HCl, 700 °C	Silica nanoparticles	62.69 nm		(311)
Teff straw	Sol–Gel method	Acid wash, 900 °C	Biosilica		>99%	(312)
Palm kernel shell ash	Sol–gel method	750 °C	Amorphous silica nanoparticle	50–98 nm		(313)

## 7 Conclusion

The increasing generation of agro-wastes, necessitated by the need to sustain a rapidly expanding global population, poses both challenges and opportunities for sustainable agricultural management. The improper disposal of these wastes, particularly through residue burning, has considerable environmental and public health consequences. At the same time, these wastes represent a valuable, underutilized resource for improving soil fertility, particularly in regions facing heavy metal and metalloid contamination such as arsenic (As) which poses significant risks to soil health and food security, especially in the rice-cultivated areas of the Ganges-Brahmaputra-Meghna plain. Arsenic not only threatens human health but also disrupts the uptake of essential plant nutrients, diminishing crop quality and exacerbating malnutrition risks.

Various remediation strategies have been explored to address these challenges, including physical, chemical, and biological approaches. This review highlights the potential of silicon (Si) and silicate-solubilizing microorganisms (SSM) in mitigating As toxicity. Si and arsenite (As^III^) utilize the same uptake transporters (Lsi1 and Lsi2), enabling Si to competitively inhibit As absorption in plants. Additionally, Si application promotes the formation of iron plaques around roots, serving as a barrier to As translocation by adsorbing or co-precipitating As in the rhizosphere. However, low solubility of Si in neutral soil makes it difficult to lessen the toxicity and buildup of As. One potential strategy is to employ consortia of silicate-solubilizing microorganisms (SSM) and agro-wastes that are rich in Si. Soil fertility is improved, the biogeochemical Si cycle is optimized, and optimal orthosilicic acid concentrations are maintained by these bio-fertilizers; as a result, agriculture can thrive even when As is present. No matter how high the As levels are, SSM tolerant to As toxicity can still promote rice development since they dissolve silicates and also increase the solubilization of phosphate and potassium. Also, agricultural residues that are rich in silicates can be bio-converted or decomposed into a bioavailable Si form more quickly with the help of SSM. This consortia based application not only mitigates As toxicity but also enhances plant resilience to biotic and abiotic stresses, and decreases dependence on expensive inorganic Si fertilizers.

The combination of SSM and Si-rich agro-wastes presents a sustainable, cost-effective, and environmentally friendly alternative to traditional remediation methods. This approach recognizes agro-wastes as a resource instead of a disposal issue, aligning with circular economy principles and enhancing environmental sustainability and public health protection.

## 8 Future perspective

Future field validation of SSM–Si agro-waste consortia across various soil types and agro-climatic areas is essential. The selection of microbial strains and the formation of consortia are necessary to improve silicate solubilisation, nutrient mobilization, and arsenic mitigation efficiency, alongside the standardization of silicon-rich agro-waste processing to provide uniform bioavailability and scalability for farm-level implementation. Comprehensive Long-term studies evaluating the effects on soil health, carbon sequestration, and agricultural yield in As-contaminated environments are necessary to corroborate laboratory findings. Pathways for the commercialization of cost-effective bioformulations accessible to resource-limited farmers should be established. Farmers will increasingly be able to adopt SSM-enriched agro-waste amendments as a low-cost alternative to conventional silicon fertilizers, thereby enhancing crop yields and nutritional quality even under arsenic-contaminated conditions. The agricultural industry can transform silicon-rich residues into standardized biofertilizer formulations, creating sustainable value chains while simultaneously reducing the harmful practice of residue burning. Policymakers and extension agencies are expected to play a critical role in mainstreaming this approach as a climate-smart, circular agriculture solution, ensuring food security while safeguarding environmental and public health.
